# Supplementary figures and images for: Sequence and structure analyses of lytic polysaccharide monooxygenases mined from metagenomic DNA of humus samples around white-rot fungi in Cuc Phuong tropical forest, Vietnam
Source: PeerJ. 2024 Jun 24;12:e17553. doi: 10.7717/peerj.17553 (PMC11210479; doi:10.7717/peerj.17553)

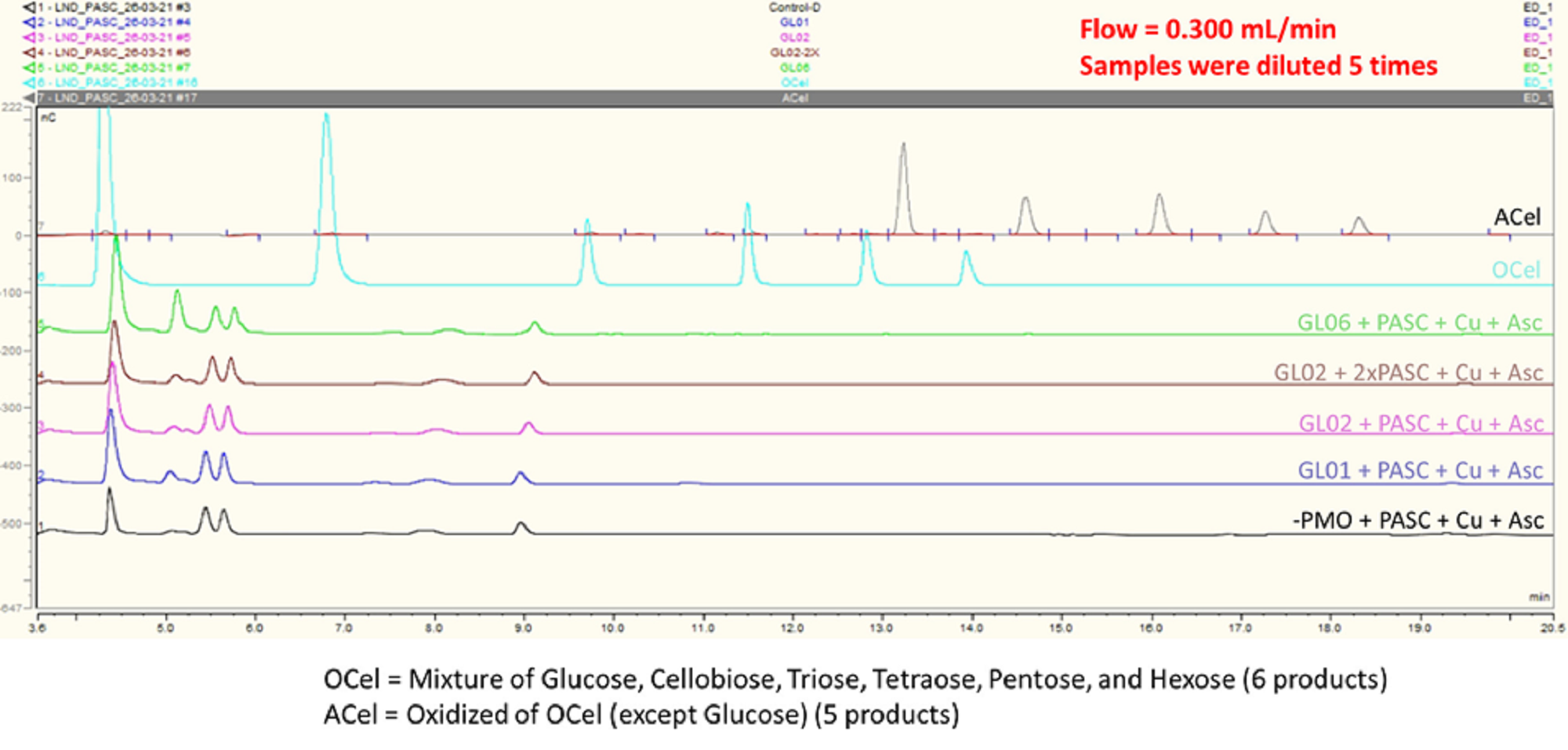

Supplement: Figure S1 — Reactions contained 5 mM GL0183513 or GL0247266, 3 mg/ml PASC, 2 mM AscA, and 10 mM CuSO_4 in 50 mM ammonium acetate buffer, pH 6.0, and were incubated at 42ôC and 800 rpm for overnight. (Control: –LPMO + PASC + Cu + AscA) [file peerj-12-17553-s001.png]
