# Supplementary material for: Sequence and structure analyses of lytic polysaccharide monooxygenases mined from metagenomic DNA of humus samples around white-rot fungi in Cuc Phuong tropical forest, Vietnam
Source: PeerJ. 2024 Jun 24;12:e17553. doi: 10.7717/peerj.17553 (PMC11210479; doi:10.7717/peerj.17553)
Supplement: Table S1 [file peerj-12-17553-s002.docx]

**Table S1. List of 31 full length amino acid sequence of putative LPMOs mined from humus samples collected around white-rot fungi of primary forest Cuc Phuong, Vietnam**

| **Group** | **query name** | **E-value** | **score** | **bias** | **stt** | **Protein Sequence** |
| --- | --- | --- | --- | --- | --- | --- |
| LPMO_10 | GL0066553 | 1,80E-42 | 132,3 | 0,4 | [Complete] | MAPTEYIKVEHLTAFSQSTVPKFAIPKFTQLSLVALALTAGSSLLSQTASAHGYVISPESRSYACKTGSNVNCGAIQWEPQSVEGPSGFPESGPADGKIASAANGAFSPLDEQSPSRWSKRDIKAGWNDFSWQFTANHVTRNWRYYLTRQGWDQNQALSRASFDLAPFCVIDGGMVQPPKLVTHNCYVPEDRTGYHVILAVWEVGDTTNSFYNAIDVNLSSGAVVPGEWTDIGDINPSLDLKAGDKVMTRVFDANGEQSAKQTQITIADATQGAKQNWPFLLASAINAQQPQLKAGQKNAAGVISPVYGKNEIFAAPKSGLERVEVSFDIAPAPGNQLNVTSLADDYTIVDGVAQVSFDVSTNADMQVSAYLFSHDGTAAGYVTQAVNNTSASFVLDVVAPKAGHYHLQVKAEPKQGEVIQQNFDLFLKDQTTAPDADFIFPEGIKSYAAGTKVLQPKTGKVYQCKPWPYSGYCVQWSPTATGFEPGVGSSWTMAWTEL |
| LPMO_10 | GL0089352 | 1,80E-30 | 93,2 | 0 | [Complete] | MNKPQTDNPLRHGRVTTPASRGAVAIDLGVLSGWQVDEMEGGKNFPALAAGPFPAPFQTDNPSVAPPADGHILSGGKTDARDCVNFTDEEMSKKLNRAFTWPLINVDAGQVFKVTWKYTAPHTTRGYRWLITKDGWDPKQRISRAQLEAQPFAEDFYPQVPYYSHAGELKAKVDHEVKLPANKKGRHVIVLMWIVANTGNAFYQAFDVDFQ |
| LPMO_10 | GL0125011 | 7,80E-41 | 127 | 0,9 | [Complete] | MQIKRRLSAEHLWALTVAAAAATVTTAAYGHGAIETPMARQYQCKLDGGQWQSSDGSSVPNAGCREAWRMLKDENPQRQFDQWNEFAAFAPNQGQSLEDVKRTVPNGLLCAGGDPTKRGMDAPQSAGFRKTEVQSGKLTMKWYATATHNPSFMRVFITKQGWRNTQPLRWEDLEPVYEGETPMPVGERYQYELQLPPGRTGDAMIYSIWQRRDAGNEGFYNCADVTFKGNGTTPPSPWHYKGPFVTPSLSPVAGDKVRFRVLTGPGKQEIVDERIDVTAQNLTSWSWTLAEAISARHGAVVQIGKRDDADVIAFDRRDIPGNASWTTDKDAVAVTSLIPGGGEPDPVAPPQAKISGPSTVKAGETITLTNEGTNNTTGQHYRYDWHAHGFTPMNGTASSWSGTAPATPGKATATLVVTDIEGRTSRATHDITITGENGGGLHPAWPAGLGTYKPGDVVTGLDGRAWACKPFPHGGWCNIAPNTPPAQWPYAPGGSGMPAAEDQRAWKLAN |
| LPMO_10 | GL0183513 | 5,20E-35 | 108 | 0 | [Complete] | MKQAVLRNGLLASSICAAIASAALVPQMASAHGYIQDPPSRDFGCQLGLNSGCGQAQYEPQSAGELPKGFPLGGPADGRIIGGNGAFSALNEQTANRWHLLPLDKHEIEFKWAYTAPHVTSKWEYFITKNDWNPNAALARASFESAPFCTIEGHGKAANDSSHPKHTCAIPADRSGHHVILGIWTVGDTGSAFYKAVDVDIQADGGPAPEWRQVSSINPHRDLKVGDEVKARAFIGGNESQEFSASISIDTVEAGLASNWSYRLAKQINDTQVLISAGVKDADGNIAPVKGSNIIFAKQESGVTNYEFAFEGSPPDAYMHLHDLKSEYVLKDGEATVGFSVMTNRSLEVTATLFDATNKQVGFQRQQVNSTTSPFAVQASSVDGEHTLKIVGKNADEEILLQEDRPLKLMTQDDAAYPTFPEGLAGYTAGTKVYAKGTTDVYECKPFPYSGYCVQWSESATQYEPGKGSHWQMAWDKH |
| LPMO_10 | GL0200824 | 2,40E-38 | 118,9 | 0 | [Complete] | MMTRKFFFSVLLMLAILVPSLIHLSAHGYVMSPASRGYQGSLDKATLGYSAAFSIYGSIINEPGSLEAPKGFPALGPADGKIASANGSIGGDTTLDIQTADRWKKTNITTGVNAFIWKYLAYHATAKWHYYMTKQGWNPNQPLSRQDLELIGTVTHNGTPPQDNVSHQITVPANRTGYHIILAVWDVADTTNAFYNVIDVNVTSGTGVSAPATPTGLTQVGVTSSSAKISWNAQTDAASYNVFRNGQNIQQVSTPTFQDTGLTANTIYTYEIQAKGSSGLMSGKSAPLNVKTNSEGTIEKPTAPSNLHSMSVTENSVSLMWMASTHSQGIKNYQIFENGIKVGETMQTNFLRTGLTQDTEYRYTVKSVAMNDQLSDASNELKIRTKKVTPGNGQSYCGAEQYNAANAYPTAGVKVFYSCKIWKNKWYANPGELPGTNMVWEEVSTCTEGPGCESSGPVTYCGAQEYNPAKTYPTAGVKVFYSFKIWENKWYANPGETPGSNAVWKVVSDCNEGSSCKSSVLTSKENNLSVMVSEHLINFTPESHYGKISRVDIITPHGLQIMTFMNPGQNSMNISRLQSGIYFVKIQYKDGNSITKTIRK |
| LPMO_10 | GL0213284 | 5,10E-42 | 130,8 | 0,1 | [Complete] | MQPRLFLAAAALLSAGSFLPGHAQAHGTLSKPMSRIYACRQDNPENPANPACAAARVIGGAQPFYDWAAINQANANGNHRAVVPDGQLCSGGNRKYRGLDLDRSDWSTTPIRADADGRYTFEFLGSAPHATREWKFYVTREGWKASDGLRWSDLEAFCTLGDVPLSEGGVYKLDCPLPRRTGQHVIYNTWQRSDSQEAFYTCADVRFEGGDVTPPPQWQDAGALTARSALEVGTTVSLRVFNVAGNDVERVDVVLASGQTAPAQWPLVLARKVNTSAARARVGILRNGVITPVASATANHVYLRPGHRFQLDTKVPDPVDPVDPPPGDYDYVYPAGIGSYVPGQTVVKGNDGKLYACRPFPEGAWCNIHAAAYNPGVGSDWRDAWIPY |
| LPMO_10 | GL0225724 | 2,30E-40 | 125,5 | 0 | [Complete] | MKKLLTGLLVAAGLLSVSLLIKTDTVSAHGYVQSPPARGYQGQLDSQSLGWTAAFNIYGNVISNPQSLEAPKGFPVSGPADGRIASANGGLGQIGDFVLDNQTSSRWKKTSISTGSNIFTWKYTAPHKTTKWHYYMTKTGWDQNAPLKRSELELIGTINHDGSPATNNLSHTINIPTDRSGYHIVLAVWDVADTSNAFYNVIDVNVNNKNSSSQVFGPFL |
| LPMO_10 | GL0247266 | 1,70E-35 | 109,6 | 0,7 | [Complete] | MLLPQKKTLAAVISSLVMSTFSGSVFAHGAMENPKARQWECHTQGGHWNPSTMGNAACKAALEGSPSKQAAFDNWNGFTGFAPGSHTVAESDVKDGMLCSGSNAGFAGFNLPHKDWTKTVLQPDSHGKVGMTYYYTAKHTPSFIEFYINKKNVDPAKKALGWNDVELLKRFDIASGDPSDRHTVNVTIPEDRTGTAVIFTRWQRIDAVGEGFYNCSDVKIKSRDGSEIPDGGGEEEGGDESDWISKGDFITAAHQPAVGEQVRFRLMGGTRGDNLVDVYLKITAQNINNNQWVLDLGRELNRDHSNELQIGQLQTDGTVRLNEQQPRANQVFVSDKGYGYAIEIVKNADAPAISLDRYTLAPIATTTSGYSYAVTGSSDKSGVTWQWKNVAGDGRITASPANQAKTQITVPGGVPAGTTATFELTGKTANGTPGKATLKVTVQAPQVAPTGPTSIASDKGGKFTAKANFDYSQGNVSYSWSLLKGSSEVSGIDQSGNVKSGLAAGDYQVKVTAELDNGERKASGTAVLKVTDKDEPGTGDYESWVAGKIYYQGDTVSWNGVNYIAGHWTTAEPGKGDNWKLHNNAKPVAWLSTMVYLMGNIVSHDGKVWKASQWIGQDLAPGQSELWKQQ |
| LPMO_10 | GL0251010 | 1,30E-34 | 106,7 | 0,3 | [Complete] | MSKLIKVSGCASSLVMMAALMTSQQVVAHGYLNDPPSRAFACQKGLNKDCGAAQYEPQSVGETAKGFPQTGVPDGKIASGALSQFAALDVQSATRWHKTEIKDRTLNFDWYYKATHPATKYEYFITRNGWNPNAALARESFDLTPFCTVDAGGKLPTDQPQGSEGPAREKHTCQIPGEKSGHHVILGIWTVHDTPGAFHDVVDVNIVAEAENPDGWRPVGNITPHADLWVGDKVKVRALTADGESAAYSSQLTIASQEDGKSENWSFKLAEQINTAQTLVRAGIRGDDGSIKPVKGANNLYAKAESGVNRFELQTELVEDSGASLSIQAFDKTVELVKGRATLPLTVLGNRTMNIEATVFDASHKAVGSATRVMEPGSATLEIALLSEPGAHQIKLIGSTTDGRTTRQRLESLELTGEGGGQAYDAVYPEGIDSYVAGTTVLQPEDGKVYECKPFPAAGWCKINGHHYKPGTGSDWQDAWILK |
| LPMO_10 | GL0293304 | 5,60E-39 | 120,9 | 0,1 | [Complete] | MSNVISKKKLQPSLRHGHIFSPKSRAYFAWEAGRMNAGQLNQREAGKFFPQLASGLRDPIAPDDQANALPPPDGRIASANQGDSIFLDEPGRHWQKHDVESHQLLTVSWHYTARHLTRRWNYFITRPDWNPDLPLSREQFEEKPFWQVQLSEQPFWSHGEALNPPNPTVHDVMLPERTGYHVLLAVWEVADTGNAFYQVIDLDFVGASVEPLPAAPAGLRATSVSTGTVALAWNAPSIPASSYRVYRDNVLITAVSGLTFNDEGLNAGTRYSYAVSSVNAAGQESARSQSIAIITLTNDAADLPPTAPTNLHSMGVTATSVSLMWGPSSSVNALQGYIVYRDGNEISRLPASQLSYLDTNVAPANSYRYFVAAIDVQGRLSVPGNVLTVTTLTQDNGGGDDNAEQGLRQWVRGTTWYTGERVRHNGLIWVCLQQHTSWLDAWAPGAVDGFTLWREAR |
| LPMO_10 | GL0297948 | 7,00E-42 | 130,4 | 3,8 | [Complete] | MQLKKLSIYTAALLFTSSALAHGYVAFPPSRAYQCNTGKNSDCGSVQWEPQSVEQASGFPEGAMPPDGQLASAGKANFSQLDSQSPTRWAKSAIKSGANNFIWHHSAPHKTTNWRYYITKQNWDQNKPLTRSDFESKPFCQIDGNGMTPAIEVTHSCNVPERTGYQVIYAVWEIADTANSFYQAIDVDFGGTGDDAENGSLWTTQLTGQLSGKDLHAGDKVIAHFFNASGEVHSLQTELTIASEAQGKSSQWSYDLAEVINTAHHDSLRAGVKDGSGNINPIYGVNQVYAPQGSALQSVTLSYSESQEDDTAKESLSLSNVAATPIENGKATVTFNAAAKGEMHLIAVVSDHAGAEKGSLQQTIDNRSLPLSIPLTNVQAGHHMLRYSASNAQGAIISQGVINLMLEEKSEPTPAPAGNYDYTFPDNLKSYTAGTKVMQPKNGKVYQCRPFPYSGYCVQWSQHASQYEPGVGSDWAMAWTEVAQ |
| LPMO_10 | GL0338092 | 8,50E-36 | 110,6 | 0 | [Complete] | MMTRKIFFPVLLILAMLVPSLVHLSAHGYVLSPASRGYQGSLDKATLGYSTAFGIYGSIINEPGSLEAPKGFPAFGPADGKIASANGSIGGDTTLDIQTADRWKKTNITTGVNAFIWKYLAYHATAKWHYYMTKQGWDPNKPLARQDLELIGTVTHNGTPPQDNVSHQITVPANRNGYHIILAVWDVADTTNAFYNVIDVNVTSGTGVAAPATPTGLAQVGVTSSSAKISWNPQSDAVSYSVFRNGQNIQQVSTPTFQDMGLIANTIYTYEIQAKGSSGLTSGKSAPLSVKTNSEGVPEKPTAPSNLHAMEVTENSASLMWTASAHSQGIKNYHVFENGIKVAETVQTTFLRTGLSQDTEYNYTVRSVGMNDQLSEVSNTLKVRTKKVTPGNGQSYCGAEPYNAANAYPIAGVKVFYACKIWKNKWYANPGEVPGTNMVWEEVSACTEGPGCESSGPVTYCGAQEYNPAKAYPTAGTKVFHACKIWENKWYANPGEAPGSNAVWKVVSDCNEGQACKTSALTHKENDLSVIVSEYMINFAPESYYNTISRVDLITPHGLQIITFTNPEKNSMNISRLQSGTYFVKILYKDGRSITKTIRK |
| LPMO_10 | GL0391320 | 2,00E-36 | 112,6 | 0 | [Complete] | MITRKIFFPVLLMLAMLVPSLIHLSAHGYVLSPASRGYQGSLDKASLGYSVAFGKYGSVINEPGSLEAPKGFPALGPADGKIASANGSIGGDTMLDLQTADRWKKTNITTGVNAFIWKYLAYHATAKWHYYMTKQGWDPNKPLSRQDLELIGTVTHNGTPPQDNVSHQIIVPANRTGYHIILAVWDVADTTNAFYNVIDVNVTSGTGVSAPATPTGLTQIGVTSSSAKMSWIPQPDAVSYAVFRNGQSIQQVSVAAFEDTGLTANTVYTYEIQAKGSSGLTSEKSTPLNIKTNSEGIQEKPTAPSNLHSMGATENSVSLMWMASTHTQGIKNYQVFENGIKVGETVQTSFSRVGLAQDTEYLYTVRSVAMNDQISDMSNELKVRTKKITPGNGQTYCGAEQYNSANAYPTAGAKVFYACKIWKNKWYANPGELPGTNMVWEEISVCTEGPGCQSSGPVTYCGAQEYNPAKTYPTAGTKVFYACKIWENKWYANSGETPGSNPVWKVIDTCTEGPGCTSGLTSKEGSLSVIVSDHMISFSPEDYYGKMSRVDVINPAGIQMLSSVNPTKSSINISHLQAGIYFVRIHYKDGSSITKTIRK |
| LPMO_10 | GL0393374 | 1,00E-30 | 94 | 0 | [Complete] | MNQPQAQTQLRHGRVISPASRGAVAIEQGLLGGWQVNEMEGGKNFPALVAGPFPAPFESDNPSVVPPADGHILSGGKDDARDCVNFTDEEMSKKLGRPFTWPLLNVTPGQTLEVKWEYTAPHTTRGYRWLITKDGWDPKQRITRAQLEAKPFAEDFYPQVPYYSHSAELKAKVNHAVKLPANKQGRHVIVLMWIVANTGNAFYQAFDVDFK |
| LPMO_10 | GL0417116 | 2,30E-42 | 132 | 2,7 | [Complete] | MKISRIALAMATLTVASSALAHGYIESPASRAYMCKQGQNIDCGLVEYEPQSVERASGFPSGSLPPDGELASAGIANYSQLDKQSQNAWTKTPMTAGVHQFVWHHTAPHKTTNWRYYITKQNWNPNKPLTRDQFDLTPFCTVNGNGQAPAMTQSMNCNVPERTGYQVIYGVWEIADTPNSFYQAIDVDFGNGGNVTPDETPAAASVWSKTLSGQVAGNNLNVGDKVIARFFDANGEIASMATELTIASAQQGDANQWAYDLAQKINSAHHDVRVGVKDESGDVSPVHGANSVFVKEDSTLKSVAISYEEQKAVVNESIAVTDLHYSKVTHGAATVTFHVNTQGDVNLEAHVLNHPGAEKGYLKQDMNNSNQDVTMNLTNVTPGHHMLKYYATNKAGTLFAQDVLDMMLEGEATSGDTGKSDFIFPDNLASYKAGTVVLQPKDGKTYECKPFPYSGYCKQYSSTSTQFEPGVGSDWKEAWMLKN |
| LPMO_10 | GL0422153 | 3,30E-36 | 111,9 | 0,5 | [Complete] | MRLEFVMHTTKTLRKSVVATGVWLAIAGAGLHIQIANAHGNLADPPSRAVLCHANQKNQNTGCGGAQYEPWSVGEAIGRFPGAGPVDGKIASGGVRSDFGALDEQAANRWHLTPIHDRNMQFDWHYQAPHPVTSWEYFITKAGWNPNAALTRASFDATPFCVVDGKHQIPAAGTGTNPKHSCTLPADRSGQHVILGVWKVGDTDKAFHSVADVDIQLDGGPAPEWPRLAEISANRDLEVGDKVTARAFDANGERTSHHVSITVANAEEGIAANWAYKLARQINETHTLVRAGQMDEDGNIEPVQGSNSIFAKAESGLTNYILDFEAAAGEPTTMHLHDVKPEYTIVDGKGGVDFSVMTNKALTVTARLFDSNNKQVSYSRQNVNGTEPFALNVVSAEGEHTLKVVGVDKRERVLLQDEKTVQLKAAGDASHDFVFPESLSSYKAGTKVLQPKNGKVYECQPFPNSGYCVQYSAGATQFEPGTGSHWNMAWTEK |
| LPMO_10 | GL0489328 | 2,20E-38 | 119 | 3,4 | [Complete] | MKLSKIALALATLTVASSALAHGYVESPASRAYMCKLGKNIDCGSVQYEPQSVERTSGFPTGALPPDAQLASAGISQYSQLDRQSLNAWTKTPISAGPNKFVWYHTAPHKTVNWRWYMTKQDWNPNKPLTRDQFESTPFCTVNGNGQAPAQRQEMNCNVPQRTGYQVIYAVWEIADTTNSFYQAIDVDFGNAGNVTPSDDTPVVVSEWNKTLDGQIAGNNVNEGDKIIARFFDANGEVTSLRTEMTVASASQGQASQWSYDLAQAINAAHSDVRVGVKDESGEVNPVHGANSVYVKEGSTLKSVAISYEEQQAQVSEDIAVTNVQFSKIKSGAATVTFHVNTQGNVNLEARVMNHAGAEKGYVKQEMNNANQDITMNLSGVTAGHHMLKYYATNKDGTLFAQDVVDMMLENDASADTGTTGKYDYTFPQSIASYKAGTVVLQPKNGKTYQCKKAPYNGYCVQYAAGANQFEPGVGSHWQMAWTLKN |
| LPMO_10 | GL0507050 | 8,10E-41 | 126,9 | 4,2 | [Complete] | MKLSKIALALATLTVASSALAHGYVESPASRAYMCKLGKNIDCGTVQYEPQSVERTSGFPTGALPPDGQLASAGISQYSQLDRQSLNAWTKTPITAGKNTFTWYHTAPHKTVNWRWYITKQDWNPNKPLTRDQFESTPFCTVNGHGQAPAQRQQMTCNVPQRTGYQVIYAVWEIADTTNSFYQAIDVDFGNGGNVTPDETPAVVSEWTNTLNGQISGNNVNAGDKVIARFFDANGEVVSMRTELTVSSASQGQASQWAFDLAQAINAAHSDVRVGMKDESGEVNPVHGANNVYVKDGSTLKSVAVSYEEQQSQVSEAIAVTNVKFSKIKNGAATVTFHVNTQGNVNLEARVMNHAGAEKGYVKQDMNNANQDVTMNLSDVTPGHHMLKYYASNKDGVMFAQDVIDMMLEGEASNTDNGTAGNYEFIFPQNIASYKAGTVVLQPKNGKTYKCKTAPYSGYCIQYAAGANQFEPGVGSHWTMAWTLKK |
| LPMO_10 | GL0522565 | 3,80E-37 | 115 | 0 | [Complete] | MNKGKTTSKGLYASSLLAAIAAAASLPQLVHAHGYVSDPPSRAYACKLGLNQQCGPVQYEPQSVGEAPKGFPASGPADGKIASGGVRGDFAAVDEQSATRWHLTSITQRELAFDWYYTIGHPATKFEYFITKTGWNANEPLTRAAFELTPFCTVDGKGELPGDGHRPGPAREKHHCTLPADRAGQHVILGAWTVHDTPAAFYNVMDVDIQIEGGTPPQWPQVGSITPHRDLRQGDKVKARAFIGTTESTQHSVSISIDNAEDGIGAHWSHKLAKRINETQPLVKAGQADAQGDIHPVLGANLIFAKAASGVTSYQLDFETKPVDDAYLHLHGIKPEYVLKDGKGAVDFSVMTNRKLQVTSQLFDEHNQQVGHASQLVDASTAPVVLEASSGAGKHLLKVVGVDNDKRVVLQEEREVRLLDAGNAEHDFVFPQSIETYRAGTLVLQGKTGEVFECKPFPAEGWCRIYSSNANQYEPGVGSHWQDAWIKR |
| LPMO_10 | GL0555809 | 4,20E-42 | 131,1 | 0,6 | [Complete] | MAATIKLNHLAAALALLASGGVLAHGYITQPESRNYLCKTGGNSQCGAIQWEPQSLEAPSGFPNGGPADGQIASAGHPQFGELDIQTSDRWTKRPVQAGPFAIGWNFTANHVTRNWRYYLTKQEWNPNQPLTRDSFDLNPFCVIDGGMVQPPKQVTHQCTLPARTGYQVVLGVWEVGDTANSFYNMIDVQFDGGTQPPLTWSQGGTIYPSIDLVAGDKARTRVFDANGERTDLQTVLTIGSAEQGQKNNWAHDLAAKINAEQSQIRAGQQTTDGQFNPVYGQNPVYLKAGSNLQRVEVQLEQQQPPASDSISVSGLESEYLLDNGKVTLAFTVTAEGDLAVTNTLYDHGGAAKGQTSADIKDSSHSFTIEVSGLSAGHHQLVIEGKPKAGGAPLQQTLDLMFKDPASGGDYQYSFPEGLKSYAAGTKVLQPKDGRIYQCKPYPYSGWCTIWTSTATQYEPGVGTNWQDAWTLVN |
| LPMO_10 | GL0600730 | 7,50E-45 | 140,1 | 0,6 | [Complete] | MNTSPRTLLSLGLLSAAMLGFSLQASAHGYVESPASRAYQCKLQLNAQCGSVQYEPQSVEGPKGFPQSGPPDGHIASANKSTFFELDQQSPARWNKLSLKTGANSFTWNLTARHSTTSWRYFITKQGWDAGQPLTRASFELTPFCQFNDGGAIPAAKVTHQCNIPADRSGSHVILAVWDVADTGNAFYQAIDVDLSK |
| LPMO_10 | GL0620585 | 2,60E-26 | 79,6 | 5,4 | [Complete] | MNNITSALAVALIGMYSTGALAHGYVFEPKSRSVIHFPPMNDKGEHSNVWPADAVEAPKLPAGNDMSPTPIANEQLFQFPPDGKLASGGSTATPAFSKLDDEKQNTYNNPMSAGPHQFKWHLPAKHRTTYFTYYITKPDWQSVPGADSRLTRAMFEDKPFCHKVYTYAPGNPSADISLPTGFETHTCDVPEREGKQKIYAVWRVRDTDNAFYQMIDVDFGGEVITKPVANVTPSSHNLSTSTLSLDGSASTGKELKYSWAVTSNADKVILENSTSAKASIRLKAKPESDFTVNVKLTVTNDKNVSDSKTVTLNAKAEADSTAPEAKAGSDFTVHSNSESRGYDLNGSASVNAEKYQWTIVSGQDIGALQVANGGEWVSTVHAAKARALIKPGKIGKVTYRLTVTAKNGKTSTDDITVTVKEAQQASDVQINTGNNQDTIVGSDVIAAAVVSKNNDSLKGAKIEWSALNHADKIQISSPNNFSTNLMGKHPYTDNVDVTLQVKVTAPDTGKVSTATKVIKLRPAQTDVKAWDHSKTYKTPCEKVSYEGNIWLNSWETRGVKPGSDGEWGVWKKEGAANMPKECK |
| LPMO_10 | GL0656018 | 1,50E-43 | 135,9 | 0 | [Complete] | MNTKFTKLRKKSLSAALLAAGIIGISTAPNAYAHGFVEKPASRAALCTQNYGALNLMCGSIMYEPQSLEAPKGFPQSGPMDGKIASAGGLFGGILDQQTTDRWFKNTITGGPNTFTWKYTAPHLTSKWHYYITKRDWNPNKPLTRADLEPIGTVAHNGSAASTNLTHTINVPTDRSGYHVILAVWDVADTSNAFYNVIDVNLVNNDGNVDTEAPTQPNGLHTTKVTSTSAELKWNPSTDNVGVKEYQVLRDGKVVATIPGTTFTDKNLNSNTKYTYSVKAVDAAGNVSTVSESLVVETLHAAPDTESPSQPTSLHTMGITSSSVDLMWGASEDNVAVDHYVIYRGSTKEQMTAVGTSTITSFKDMNLQSNTSYIYAVAAVDVAGNESIKSSILTVTTKEQSTSYEQWSPFKAYKKGDKVEYQGKNYEAVQSYQGYGDPNWINALSLWKEIK |
| LPMO_10 | GL0658692 | 6,30E-40 | 124 | 0,5 | [Complete] | MHLFHRAVLALSVVAGLALSGSAFAHGTMTKPLSRVKQCHEGNPENPTNPACAAAKAIGGSQPFYDWSAIAHGNANGNHRELVRDGELCSASIPKYRGLDLNRTDWPTTPIRADARGRYTFEFRAPAPHATREWKFYVTRDGWQPGSPLRWADLQEFCTLGNTPLSEGGVYKLDCPLPKRGGQHIIYNTWQRSDSLEAFYTCMDVRFEGGDGVVPPPQWQDAGAVTANGALDVDSTVTLRVFNANGNDLERVETTLAAGQTAPAQWPLALARKVNASAQHARVGVMKNGVIAPVASATENRVYLKPGNRFQLETQVPGPVDPVDPPGGDFDFVYPAGLGSYTPGETVVKGTDGKLYACRPFPEGAWCNINADAYRPGTGYAWRDAWVAY |
| LPMO_10 | GL0681738 | 2,70E-30 | 92,6 | 0 | [Complete] | MNQPQAQTQLRHGRVISPASRGAVAIEQGLLGGWQVNEMEGGKNFPALAAGPFPAPFESDNPSVVPPADGYILSGGKNDARDCVNFTAEEMSKKLGRPFTWPLLNVTPGQTLEVKWEYTAPHTTRGYRWLITKDGWDPKQRITRAQLEAQPFAEDFYPQVPYYSHSAELKAKVNHAVKLPGNKQGHHVIVLMWIVANTGNAFYQAFDVDFK |
| LPMO_10 | GL0772141 | 8,10E-43 | 133,5 | 6,7 | [Complete] | MKLSKIALAVAALTVASSAFAHGYIESPASRAYMCKLGHNIDCGSVQYEPQSVEKASGFPTGAMPPDGQLASAGIANYSQLDKQSLNAWTKNPMTAGPHEFVWHHTAPHKTTNWRYYITKQNWDPNKPLTRDQFELTPFCTINGNGQAPAMTKSMTCNVPERTGYQVIYGVWEIADTANSFYQAIDVDFGNGGNVTPDDTPAVISQWSKTLSGQVAGNNLNTGDKVIARFFDANGEVAALRTELNIGSAAQGDANQWSYDLAQKINAAHSDMRVGVKDEAGEISPVHGANSVFVKDGSTLQSVAISYEEQKAEVHETIAVSNLQYSNIENGKATVTFHVNTKGDVNVEAHVMSHSGAEKGYLKQEMNNANQDVTMTLTDVTAGHHMLKYYASNKDGTLFDQDVLNLMLENNAADSTGQHDVIFPDGIASYKAGTVVLQPKDGKTYECKPFPYSGYCVQYSPTANQFEPGVGTHWKEAWVLKN |
| LPMO_10 | GL0875000 | 1,30E-39 | 123 | 2,5 | [Complete] | MKLSKIALALATLTVASSALAHGYVDTPASRAYMCKLGKNIDCGTVQYEPQSVERTSGFPAGALPPDGQLASAGISQYSQLDRQSLNAWTKTPITAGPNKFVWYHTAPHKTVNWRWYITKQDWNPNKPLTRDQLEPTPFCTVNGNGQAPAQRQEMNCNVPQRTGYQVIYAVWEIADTSNSFYQAIDVDFGNGGNVTPDETPAVVSEWNKTLDGQISGNNVNAGDKVIARFFDANGEVASMRTELTVSSASQGNASQWSYDLAQAINAAHSDVRVGTKDESGVVNPVHGVNNVYVKDGSTLKSVAISYEEQQAQVSENIAVSNVKFSKIANGNATVTFHVNTQGNVNLEARVMNHAGAEKGYLKQDMNNANQDVTLNLTDVTPGHHMLKYYAVNKDGTLFAQDVVDMMLEGEAASGNTGNTGNFDYTFPENIKSYKAGTVVLQPKDGKTYKCKNAPYSGYCVQYAQNANQFEPGVGSHWQMAWTVK |
| LPMO_10 | GL0883009 | 2,10E-33 | 102,8 | 0,2 | [Complete] | MSLKKPDSARDPQAISREVAPRHGAVSSPKSRAYFAAEKGLIEGNLQYGIEWGKFFPAVTGGLKDPYASTDVANIAPPVDGKIASGERPGATYLDRTDVDWQKHKVYGGEALDFVWRFVALHKYRRFNYFITRVCWDPSQPLSRAQFEAEPFATFLNTRQPYWSYTDAEMWPANPTTHTLTLPNREGYYVLLGVWEVAETDKAFYQVIDLDFLPGGEGPDGPDAPTELHDVGTTETSVDLRWTAAQSGGPIDGFIIYRDGNEVGRVPASQRTFTDDNLTPDTRYRYTVKAFDAQGQLSASSNVHDAHTKPKEDGGDYRDWKLKDSYTTGEVVRHNQTLWQCLASHTAHVESWAPGAADGFTLWKEYVGRR |
| LPMO_10 | GL0999597 | 6,40E-41 | 127,3 | 0,5 | [Complete] | MKLSKIVLALATLGMAGGALAHGYVSQPASRAFMCNANMGSQNKDCGSGPLYEMQSVEGADGFPELNNGPEDGHIASAGVASMSRLDEQSETRWVKHPISAGKQSFTWTYTAPHKTKSWDYYLTKSDWNPAQPLTRGSFESKPFCHIEGNNIRPSAGPDMPHECTVPEREGYHVILAAWDVADTGATFYNVIDVDFGGHNAPAPAPDEDPQSDLPAQWHSDTVYVGGDRVTWNGKEYQARWWTRGNQPGVHEVWSEVK |
| LPMO_10 | GL1004230 | 1,60E-35 | 109,6 | 0,6 | [Complete] | MGAHWLLFIFITKDCESNEMPERDARTGPLPFMRSFWDSKMSRTPLFWKFSARLALAAYGICCVATSYGHGRVTEPPSRIVLCTMNDNADCYVDAWHANAMEKGKFFPATQAGLADVFARDDARNDQPPDDGRIAGSSVNGDLPVLNEQTPRRWKKNAVRSGAMQSFTWEYSAIHKTRRWNYFITKSGWNASAPLTRAQFEEKPFCTVQNAGQPYWSPSADLMPKNPTIHQCRLPERSGYQVILAVWEVADTPMGFYQVIDTEFVSGNMGDVVSPFGTK |
| LPMO_10 | GL1034380 | 1,00E-36 | 113,6 | 0,1 | [Complete] | MNHRAKIVRIGLLALLIAAAVGALLARKVPLVAPAPQARMTNAPQHGAMEMPIARQLMCYEAGGFYWPYDGSGIRDAGCRAAYQYVYAKFQDDAQQAAYQFEQWHEVSTNVADYNNPEAVKAAIPDGTLCSAGNVLDNQDIPAVLQRVNEFINELHGRAVRISVRINDKSGLDRPAPWPTQTLTKNSENKATLTYRIATPHSPSFWEIYISRPGYDPTREPLTWADLEAPQRLEEVPAVDGKYELQVDLGNHSGRRVIYSRWQRVDAAGEGFYNCSDVNIVAGP |
