# Supplementary material for: Sequence and structure analyses of lytic polysaccharide monooxygenases mined from metagenomic DNA of humus samples around white-rot fungi in Cuc Phuong tropical forest, Vietnam
Source: PeerJ. 2024 Jun 24;12:e17553. doi: 10.7717/peerj.17553 (PMC11210479; doi:10.7717/peerj.17553)
Supplement: Table S2 [file peerj-12-17553-s003.docx]

**Table S2. Protein identification by MS/MS analysis**

| **Protein Group** | **Protein ID** | **Accession** | **-10lgP** | **Coverage (%)** | **#Peptides** | **#Unique** | **PTM** | **Mass** | **Description** |
| --- | --- | --- | --- | --- | --- | --- | --- | --- | --- |
| 1 | 1 | **P0A6Z3\|HTPG_ECOLI** | 348,99 | 29 | 14 | 14 | N | 71423 | **Chaperone protein HtpG OS=Escherichia coli (strain K12) OX=83333 GN=htpG PE=1 SV=1** |
| 8 | 2 | B1X8W8\|ARNA_ECODH | 162,19 | 7 | 4 | 4 | N | 74289 | Bifunctional polymyxin resistance protein ArnA OS=Escherichia coli (strain K12 / DH10B) OX=316385 GN=arnA PE=3 SV=1 |
| 8 | 3 | C4ZU97\|ARNA_ECOBW | 162,19 | 7 | 4 | 4 | N | 74289 | Bifunctional polymyxin resistance protein ArnA OS=Escherichia coli (strain K12 / MC4100 / BW2952) OX=595496 GN=arnA PE=3 SV=1 |
| 8 | 4 | P77398\|ARNA_ECOLI | 162,19 | 7 | 4 | 4 | N | 74289 | Bifunctional polymyxin resistance protein ArnA OS=Escherichia coli (strain K12) OX=83333 GN=arnA PE=1 SV=1 |
| 73 | 15 | P46853\|YHHX_ECOLI | 118,79 | 7 | 2 | 2 | N | 38765 | Uncharacterized oxidoreductase YhhX OS=Escherichia coli (strain K12) OX=83333 GN=yhhX PE=1 SV=1 |
| 38 | 6 | P0CE48\|EFTU2_ECOLI | 68,41 | 7 | 2 | 2 | N | 43314 | Elongation factor Tu 2 OS=Escherichia coli (strain K12) OX=83333 GN=tufB PE=1 SV=1 |
| 38 | 7 | tr\|A0A0M3KKV1\|A0A0M3KKV1_ECODH | 68,41 | 7 | 2 | 2 | N | 43284 | Elongation factor Tu OS=Escherichia coli (strain K12 / DH10B) OX=316385 GN=tufA PE=1 SV=1 |
| 38 | 8 | P0CE47\|EFTU1_ECOLI | 68,41 | 7 | 2 | 2 | N | 43284 | Elongation factor Tu 1 OS=Escherichia coli (strain K12) OX=83333 GN=tufA PE=1 SV=1 |
| 344 | 143 | P30871\|3PASE_ECOLI | 53,53 | 3 | 1 | 1 | N | 48389 | Inorganic triphosphatase OS=Escherichia coli (strain K12) OX=83333 GN=ygiF PE=1 SV=1 |
| 46 | 9 | P0A6M8\|EFG_ECOLI | 53,44 | 4 | 2 | 2 | Y | 77581 | Elongation factor G OS=Escherichia coli (strain K12) OX=83333 GN=fusA PE=1 SV=2 |
| 46 | 10 | B1X6J0\|EFG_ECODH | 53,44 | 4 | 2 | 2 | Y | 77581 | Elongation factor G OS=Escherichia coli (strain K12 / DH10B) OX=316385 GN=fusA PE=3 SV=1 |
| 46 | 11 | C4ZUJ5\|EFG_ECOBW | 53,44 | 4 | 2 | 2 | Y | 77581 | Elongation factor G OS=Escherichia coli (strain K12 / MC4100 / BW2952) OX=595496 GN=fusA PE=3 SV=1 |
| 345 | 185 | C4ZPU1\|DNAJ_ECOBW | 45,83 | 3 | 1 | 1 | Y | 41128 | Chaperone protein DnaJ OS=Escherichia coli (strain K12 / MC4100 / BW2952) OX=595496 GN=dnaJ PE=3 SV=1 |
| 345 | 186 | P08622\|DNAJ_ECOLI | 45,83 | 3 | 1 | 1 | Y | 41100 | Chaperone protein DnaJ OS=Escherichia coli (strain K12) OX=83333 GN=dnaJ PE=1 SV=3 |
| 345 | 187 | B1XBE0\|DNAJ_ECODH | 45,83 | 3 | 1 | 1 | Y | 41100 | Chaperone protein DnaJ OS=Escherichia coli (strain K12 / DH10B) OX=316385 GN=dnaJ PE=3 SV=1 |
| 2 | 124 | Q46939\|YQEF_ECOLI | 43,92 | 2 | 2 | 2 | N | 41019 | Probable acetyl-CoA acetyltransferase OS=Escherichia coli (strain K12) OX=83333 GN=yqeF PE=3 SV=2 |
| 347 | 191 | P46130\|YBHC_ECOLI | 43,33 | 2 | 1 | 1 | N | 46082 | Putative acyl-CoA thioester hydrolase YbhC OS=Escherichia coli (strain K12) OX=83333 GN=ybhC PE=1 SV=2 |
| 57 | 47 | tr\|A0A8A4RZ47\|A0A8A4RZ47_ECOLI | 41,33 | 3 | 2 | 2 | N | 92591 | Protein TraC OS=Escherichia coli str. K-12 substr. MG1655 OX=511145 GN=traC PE=4 SV=1 |
| 5 | 21 | tr\|A0A8A4RYE2\|A0A8A4RYE2_ECOLI | 40,99 | 3 | 5 | 5 | N | 200718 | DUF4165 domain-containing protein OS=Escherichia coli str. K-12 substr. MG1655 OX=511145 GN=ICJKHHIM_00060 PE=4 SV=1 |
| 348 | 278 | P00370\|DHE4_ECOLI | 40,31 | 5 | 1 | 1 | N | 48581 | NADP-specific glutamate dehydrogenase OS=Escherichia coli (strain K12) OX=83333 GN=gdhA PE=1 SV=1 |
| 349 | 280 | P0AFP9\|YBHR_ECOLI | 40,19 | 2 | 1 | 1 | N | 41566 | Probable multidrug ABC transporter permease YbhR OS=Escherichia coli (strain K12) OX=83333 GN=ybhR PE=1 SV=1 |
| 25 | 17 | P32674\|GRE1_ECOLI | 39,77 | 3 | 2 | 2 | N | 85960 | Probable dehydratase PflD OS=Escherichia coli (strain K12) OX=83333 GN=pflD PE=3 SV=1 |
| 15 | 18 | P11454\|ENTF_ECOLI | 39,28 | 4 | 3 | 3 | N | 141991 | Enterobactin synthase component F OS=Escherichia coli (strain K12) OX=83333 GN=entF PE=1 SV=3 |
| 18 | 20 | P37676\|YIAO_ECOLI | 39,23 | 6 | 3 | 3 | N | 35970 | 2_3-diketo-L-gulonate-binding periplasmic protein YiaO OS=Escherichia coli (strain K12) OX=83333 GN=yiaO PE=1 SV=1 |
| 350 | 283 | P76113\|CURA_ECOLI | 38,45 | 4 | 1 | 1 | Y | 37610 | NADPH-dependent curcumin reductase OS=Escherichia coli (strain K12) OX=83333 GN=curA PE=1 SV=3 |
| 350 | 284 | tr\|B1XDG0\|B1XDG0_ECODH | 38,45 | 4 | 1 | 1 | Y | 37610 | NADPH-dependent curcumin/dihydrocurcumin reductase OS=Escherichia coli (strain K12 / DH10B) OX=316385 GN=curA PE=4 SV=1 |
| 39 | 24 | P06959\|ODP2_ECOLI | 37,18 | 4 | 2 | 2 | N | 66096 | Dihydrolipoyllysine-residue acetyltransferase component of pyruvate dehydrogenase complex OS=Escherichia coli (strain K12) OX=83333 GN=aceF PE=1 SV=3 |
| 9 | 221 | P77783\|YNFF_ECOLI | 36,19 | 1 | 1 | 1 | N | 89987 | Probable dimethyl sulfoxide reductase chain YnfF OS=Escherichia coli (strain K12) OX=83333 GN=ynfF PE=1 SV=4 |
| 21 | 294 | P25741\|RFAP_ECOLI | 35,92 | 3 | 1 | 1 | N | 30872 | Lipopolysaccharide core heptose(I) kinase RfaP OS=Escherichia coli (strain K12) OX=83333 GN=rfaP PE=3 SV=1 |
| 351 | 299 | P23878\|FEPC_ECOLI | 35,76 | 3 | 1 | 1 | N | 29784 | Ferric enterobactin transport ATP-binding protein FepC OS=Escherichia coli (strain K12) OX=83333 GN=fepC PE=1 SV=2 |
| 59 | 118 | P07001\|PNTA_ECOLI | 34,94 | 7 | 2 | 2 | N | 54623 | NAD(P) transhydrogenase subunit alpha OS=Escherichia coli (strain K12) OX=83333 GN=pntA PE=1 SV=2 |
| 74 | 271 | tr\|Q2A0K9\|Q2A0K9_ECOLI | 34,78 | 7 | 2 | 2 | Y | 53482 | Exodeoxyribonuclease I OS=Escherichia coli (strain K12) OX=83333 GN=sbcB PE=4 SV=1 |
| 74 | 272 | P04995\|EX1_ECOLI | 34,78 | 7 | 2 | 2 | Y | 54501 | Exodeoxyribonuclease I OS=Escherichia coli (strain K12) OX=83333 GN=sbcB PE=1 SV=2 |
| 74 | 273 | tr\|A0A0G3HGF9\|A0A0G3HGF9_ECOLI | 34,78 | 7 | 2 | 2 | Y | 54501 | Exodeoxyribonuclease I OS=Escherichia coli (strain K12) OX=83333 GN=sbcB PE=4 SV=1 |
| 74 | 274 | tr\|Q2A0L0\|Q2A0L0_ECOLI | 34,78 | 7 | 2 | 2 | Y | 54529 | Exodeoxyribonuclease I OS=Escherichia coli (strain K12) OX=83333 GN=sbcB PE=4 SV=1 |
| 355 | 458 | B1XE99\|LSRK_ECODH | 34,63 | 1 | 1 | 1 | N | 57545 | Autoinducer-2 kinase OS=Escherichia coli (strain K12 / DH10B) OX=316385 GN=lsrK PE=3 SV=1 |
| 355 | 459 | P77432\|LSRK_ECOLI | 34,63 | 1 | 1 | 1 | N | 57545 | Autoinducer-2 kinase OS=Escherichia coli (strain K12) OX=83333 GN=lsrK PE=1 SV=1 |
| 230 | 317 | P00579\|RPOD_ECOLI | 34,59 | 1 | 1 | 1 | N | 70263 | RNA polymerase sigma factor RpoD OS=Escherichia coli (strain K12) OX=83333 GN=rpoD PE=1 SV=2 |
| 75 | 275 | P14377\|ZRAS_ECOLI | 34,32 | 7 | 2 | 2 | N | 51032 | Sensor protein ZraS OS=Escherichia coli (strain K12) OX=83333 GN=zraS PE=1 SV=2 |
| 222 | 242 | P31471\|YIEL_ECOLI | 33,6 | 2 | 1 | 1 | N | 42755 | Uncharacterized protein YieL OS=Escherichia coli (strain K12) OX=83333 GN=yieL PE=4 SV=3 |
| 76 | 281 | Q59385\|COPA_ECOLI | 33,45 | 3 | 2 | 2 | N | 87873 | Copper-exporting P-type ATPase OS=Escherichia coli (strain K12) OX=83333 GN=copA PE=1 SV=4 |
| 62 | 167 | P33136\|OPGG_ECOLI | 33,41 | 4 | 2 | 2 | N | 57913 | Glucans biosynthesis protein G OS=Escherichia coli (strain K12) OX=83333 GN=mdoG PE=1 SV=1 |
| 62 | 168 | C4ZRY9\|OPGG_ECOBW | 33,41 | 4 | 2 | 2 | N | 57913 | Glucans biosynthesis protein G OS=Escherichia coli (strain K12 / MC4100 / BW2952) OX=595496 GN=mdoG PE=3 SV=1 |
| 62 | 169 | B1X9G2\|OPGG_ECODH | 33,41 | 4 | 2 | 2 | N | 57913 | Glucans biosynthesis protein G OS=Escherichia coli (strain K12 / DH10B) OX=316385 GN=mdoG PE=3 SV=1 |
| 124 | 94 | P32051\|YDEK_ECOLI | 32,46 | 2 | 1 | 1 | N | 136514 | Uncharacterized lipoprotein YdeK OS=Escherichia coli (strain K12) OX=83333 GN=ydeK PE=5 SV=2 |
| 26 | 25 | P0C7L2\|PAAJ_ECOLI | 32 | 10 | 2 | 2 | N | 42277 | 3-oxoadipyl-CoA/3-oxo-5_6-dehydrosuberyl-CoA thiolase OS=Escherichia coli (strain K12) OX=83333 GN=paaJ PE=1 SV=1 |
| 60 | 123 | P38104\|RSPA_ECOLI | 31,99 | 9 | 2 | 2 | N | 45968 | Starvation-sensing protein RspA OS=Escherichia coli (strain K12) OX=83333 GN=rspA PE=2 SV=1 |
| 82 | 437 | P39333\|BDCA_ECOLI | 31,54 | 8 | 1 | 1 | Y | 24598 | Cyclic-di-GMP-binding biofilm dispersal mediator protein OS=Escherichia coli (strain K12) OX=83333 GN=bdcA PE=1 SV=2 |
| 22 | 484 | P76237\|DGCJ_ECOLI | 30,73 | 3 | 1 | 1 | Y | 56603 | Probable diguanylate cyclase DgcJ OS=Escherichia coli (strain K12) OX=83333 GN=dgcJ PE=1 SV=2 |
| 36 | 93 | P06612\|TOP1_ECOLI | 29,5 | 4 | 2 | 2 | Y | 97350 | DNA topoisomerase 1 OS=Escherichia coli (strain K12) OX=83333 GN=topA PE=1 SV=2 |
| 58 | 110 | P0AEM9\|TCYJ_ECOLI | 29,12 | 17 | 2 | 2 | N | 29039 | L-cystine-binding protein TcyJ OS=Escherichia coli (strain K12) OX=83333 GN=tcyJ PE=1 SV=1 |
| 27 | 117 | P38054\|CUSA_ECOLI | 29,08 | 3 | 2 | 2 | N | 114707 | Cation efflux system protein CusA OS=Escherichia coli (strain K12) OX=83333 GN=cusA PE=1 SV=2 |
| 52 | 68 | P0AF06\|MOTB_ECOLI | 28,84 | 12 | 2 | 2 | N | 34186 | Motility protein B OS=Escherichia coli (strain K12) OX=83333 GN=motB PE=1 SV=1 |
| 357 | 529 | tr\|A0A8A4RYR7\|A0A8A4RYR7_ECOLI | 28,24 | 5 | 1 | 1 | N | 35095 | Peptidase S49 domain-containing protein OS=Escherichia coli str. K-12 substr. MG1655 OX=511145 GN=ICJKHHIM_00152 PE=3 SV=1 |
| 40 | 40 | P04993\|RECD_ECOLI | 28,16 | 3 | 2 | 2 | N | 66902 | RecBCD enzyme subunit RecD OS=Escherichia coli (strain K12) OX=83333 GN=recD PE=1 SV=2 |
| 29 | 64 | P37650\|BCSC_ECOLI | 28,01 | 3 | 2 | 2 | N | 127724 | Cellulose synthase operon protein C OS=Escherichia coli (strain K12) OX=83333 GN=bcsC PE=1 SV=3 |
| 360 | 534 | P37902\|GLTI_ECOLI | 27,55 | 8 | 1 | 1 | N | 33420 | Glutamate/aspartate import solute-binding protein OS=Escherichia coli (strain K12) OX=83333 GN=gltI PE=1 SV=2 |
| 13 | 37 | P0AFI2\|PARC_ECOLI | 27,4 | 4 | 2 | 2 | Y | 83831 | DNA topoisomerase 4 subunit A OS=Escherichia coli (strain K12) OX=83333 GN=parC PE=1 SV=1 |
| 71 | 538 | P39406\|RSMC_ECOLI | 26,67 | 6 | 1 | 1 | N | 37625 | Ribosomal RNA small subunit methyltransferase C OS=Escherichia coli (strain K12) OX=83333 GN=rsmC PE=1 SV=3 |
| 71 | 539 | B1XFI0\|RSMC_ECODH | 26,67 | 6 | 1 | 1 | N | 37625 | Ribosomal RNA small subunit methyltransferase C OS=Escherichia coli (strain K12 / DH10B) OX=316385 GN=rsmC PE=3 SV=1 |
| 363 | 542 | P0A7V3\|RS3_ECOLI | 26,49 | 7 | 1 | 1 | N | 25983 | 30S ribosomal protein S3 OS=Escherichia coli (strain K12) OX=83333 GN=rpsC PE=1 SV=2 |
| 363 | 543 | C4ZUG9\|RS3_ECOBW | 26,49 | 7 | 1 | 1 | N | 25983 | 30S ribosomal protein S3 OS=Escherichia coli (strain K12 / MC4100 / BW2952) OX=595496 GN=rpsC PE=3 SV=1 |
| 34 | 54 | P33666\|YDBA_ECOLI | 26,37 | 5 | 2 | 2 | Y | 87130 | Exported protein YdbA OS=Escherichia coli (strain K12) OX=83333 GN=ydbA PE=3 SV=3 |
| 54 | 173 | P27247\|PLSX_ECOLI | 26,17 | 14 | 2 | 2 | N | 38214 | Phosphate acyltransferase OS=Escherichia coli (strain K12) OX=83333 GN=plsX PE=1 SV=2 |
| 54 | 174 | C4ZS30\|PLSX_ECOBW | 26,17 | 14 | 2 | 2 | N | 38214 | Phosphate acyltransferase OS=Escherichia coli (strain K12 / MC4100 / BW2952) OX=595496 GN=plsX PE=3 SV=1 |
| 54 | 175 | B1XA00\|PLSX_ECODH | 26,17 | 14 | 2 | 2 | N | 38214 | Phosphate acyltransferase OS=Escherichia coli (strain K12 / DH10B) OX=316385 GN=plsX PE=3 SV=1 |
| 228 | 310 | P37647\|KDGK_ECOLI | 25,81 | 6 | 1 | 1 | N | 33962 | 2-dehydro-3-deoxygluconokinase OS=Escherichia coli (strain K12) OX=83333 GN=kdgK PE=1 SV=1 |
| 365 | 546 | P77171\|YDCI_ECOLI | 25,74 | 3 | 1 | 1 | N | 33402 | Uncharacterized HTH-type transcriptional regulator YdcI OS=Escherichia coli (strain K12) OX=83333 GN=ydcI PE=3 SV=2 |
| 368 | 552 | P76186\|YDHK_ECOLI | 25,2 | 1 | 1 | 1 | N | 75267 | Uncharacterized transporter YdhK OS=Escherichia coli (strain K12) OX=83333 GN=ydhK PE=3 SV=1 |
| 145 | 151 | P76578\|A2MG_ECOLI | 25,18 | 1 | 1 | 1 | N | 181584 | Alpha-2-macroglobulin OS=Escherichia coli (strain K12) OX=83333 GN=yfhM PE=1 SV=1 |
| 98 | 34 | C5A0S7\|RPOB_ECOBW | 25,17 | 1 | 1 | 1 | N | 150632 | DNA-directed RNA polymerase subunit beta OS=Escherichia coli (strain K12 / MC4100 / BW2952) OX=595496 GN=rpoB PE=3 SV=1 |
| 98 | 35 | P0A8V2\|RPOB_ECOLI | 25,17 | 1 | 1 | 1 | N | 150632 | DNA-directed RNA polymerase subunit beta OS=Escherichia coli (strain K12) OX=83333 GN=rpoB PE=1 SV=1 |
| 98 | 36 | B1XBY9\|RPOB_ECODH | 25,17 | 1 | 1 | 1 | N | 150632 | DNA-directed RNA polymerase subunit beta OS=Escherichia coli (strain K12 / DH10B) OX=316385 GN=rpoB PE=3 SV=1 |
| 65 | 255 | P0AG30\|RHO_ECOLI | 25,16 | 8 | 2 | 2 | N | 47004 | Transcription termination factor Rho OS=Escherichia coli (strain K12) OX=83333 GN=rho PE=1 SV=1 |
| 56 | 404 | P0ADW6\|YHCC_ECOLI | 25,11 | 8 | 2 | 2 | Y | 34607 | Protein YhcC OS=Escherichia coli (strain K12) OX=83333 GN=yhcC PE=3 SV=1 |
| 64 | 238 | P09424\|MTLD_ECOLI | 25,01 | 7 | 2 | 2 | N | 41139 | Mannitol-1-phosphate 5-dehydrogenase OS=Escherichia coli (strain K12) OX=83333 GN=mtlD PE=1 SV=3 |
| 64 | 239 | C4ZXJ1\|MTLD_ECOBW | 25,01 | 7 | 2 | 2 | N | 41139 | Mannitol-1-phosphate 5-dehydrogenase OS=Escherichia coli (strain K12 / MC4100 / BW2952) OX=595496 GN=mtlD PE=3 SV=1 |
| 64 | 240 | B1X8L4\|MTLD_ECODH | 25,01 | 7 | 2 | 2 | N | 41139 | Mannitol-1-phosphate 5-dehydrogenase OS=Escherichia coli (strain K12 / DH10B) OX=316385 GN=mtlD PE=3 SV=1 |
| 64 | 241 | tr\|Q0PWM5\|Q0PWM5_ECOW3 | 25,01 | 7 | 2 | 2 | N | 41113 | Mannitol-1-phosphate 5-dehydrogenase OS=Escherichia coli (strain K12 / W3110 / ATCC 27325 / DSM 5911) OX=316407 GN=mtlD PE=3 SV=1 |
| 132 | 57 | P76393\|YEGI_ECOLI | 24,72 | 1 | 1 | 1 | N | 71639 | Protein kinase YegI OS=Escherichia coli (strain K12) OX=83333 GN=yegI PE=1 SV=1 |
| 170 | 56 | P0AD61\|KPYK1_ECOLI | 24,72 | 4 | 1 | 1 | Y | 50729 | Pyruvate kinase I OS=Escherichia coli (strain K12) OX=83333 GN=pykF PE=1 SV=1 |
| 80 | 409 | P07813\|SYL_ECOLI | 24,67 | 3 | 2 | 2 | Y | 97234 | Leucine--tRNA ligase OS=Escherichia coli (strain K12) OX=83333 GN=leuS PE=1 SV=2 |
| 80 | 410 | C4ZWC9\|SYL_ECOBW | 24,67 | 3 | 2 | 2 | Y | 97234 | Leucine--tRNA ligase OS=Escherichia coli (strain K12 / MC4100 / BW2952) OX=595496 GN=leuS PE=3 SV=1 |
| 371 | 555 | P77211\|CUSC_ECOLI | 24,57 | 2 | 1 | 1 | N | 50270 | Cation efflux system protein CusC OS=Escherichia coli (strain K12) OX=83333 GN=cusC PE=1 SV=1 |
| 282 | 559 | P64614\|YHCN_ECOLI | 24,29 | 16 | 1 | 1 | N | 9196 | Uncharacterized protein YhcN OS=Escherichia coli (strain K12) OX=83333 GN=yhcN PE=3 SV=1 |
| 290 | 589 | P36547\|EUTR_ECOLI | 24,12 | 2 | 1 | 1 | N | 40160 | HTH-type DNA-binding transcriptional activator EutR OS=Escherichia coli (strain K12) OX=83333 GN=eutR PE=3 SV=2 |
| 83 | 560 | P77257\|LSRA_ECOLI | 24,03 | 2 | 1 | 1 | N | 55821 | Autoinducer 2 import ATP-binding protein LsrA OS=Escherichia coli (strain K12) OX=83333 GN=lsrA PE=1 SV=1 |
| 83 | 561 | B1XEA1\|LSRA_ECODH | 24,03 | 2 | 1 | 1 | N | 55821 | Autoinducer 2 import ATP-binding protein LsrA OS=Escherichia coli (strain K12 / DH10B) OX=316385 GN=lsrA PE=3 SV=1 |
| 240 | 403 | P0AFV8\|PSPD_ECOLI | 23,94 | 8 | 1 | 1 | N | 8042 | Phage shock protein D OS=Escherichia coli (strain K12) OX=83333 GN=pspD PE=2 SV=1 |
| 81 | 428 | B1XB97\|HSLU_ECODH | 23,59 | 8 | 2 | 2 | Y | 49594 | ATP-dependent protease ATPase subunit HslU OS=Escherichia coli (strain K12 / DH10B) OX=316385 GN=hslU PE=3 SV=1 |
| 81 | 429 | C5A098\|HSLU_ECOBW | 23,59 | 8 | 2 | 2 | Y | 49594 | ATP-dependent protease ATPase subunit HslU OS=Escherichia coli (strain K12 / MC4100 / BW2952) OX=595496 GN=hslU PE=3 SV=1 |
| 81 | 430 | P0A6H5\|HSLU_ECOLI | 23,59 | 8 | 2 | 2 | Y | 49594 | ATP-dependent protease ATPase subunit HslU OS=Escherichia coli (strain K12) OX=83333 GN=hslU PE=1 SV=1 |
| 229 | 316 | P45804\|YHGE_ECOLI | 23,56 | 2 | 1 | 1 | N | 64637 | Uncharacterized protein YhgE OS=Escherichia coli (strain K12) OX=83333 GN=yhgE PE=4 SV=1 |
| 374 | 564 | P0A7U3\|RS19_ECOLI | 23,49 | 16 | 1 | 1 | N | 10430 | 30S ribosomal protein S19 OS=Escherichia coli (strain K12) OX=83333 GN=rpsS PE=1 SV=2 |
| 374 | 565 | B1X6G7\|RS19_ECODH | 23,49 | 16 | 1 | 1 | N | 10430 | 30S ribosomal protein S19 OS=Escherichia coli (strain K12 / DH10B) OX=316385 GN=rpsS PE=3 SV=1 |
| 374 | 566 | C4ZUH1\|RS19_ECOBW | 23,49 | 16 | 1 | 1 | N | 10430 | 30S ribosomal protein S19 OS=Escherichia coli (strain K12 / MC4100 / BW2952) OX=595496 GN=rpsS PE=3 SV=1 |
| 131 | 39 | P39321\|TAMB_ECOLI | 23,45 | 1 | 1 | 1 | N | 136780 | Translocation and assembly module subunit TamB OS=Escherichia coli (strain K12) OX=83333 GN=tamB PE=1 SV=2 |
| 227 | 300 | P0AB46\|YMGD_ECOLI | 23,34 | 13 | 1 | 1 | Y | 11852 | Uncharacterized protein YmgD OS=Escherichia coli (strain K12) OX=83333 GN=ymgD PE=1 SV=2 |
| 147 | 163 | P0A8T7\|RPOC_ECOLI | 23,14 | 1 | 1 | 1 | N | 155160 | DNA-directed RNA polymerase subunit beta' OS=Escherichia coli (strain K12) OX=83333 GN=rpoC PE=1 SV=1 |
| 147 | 164 | B1XBZ0\|RPOC_ECODH | 23,14 | 1 | 1 | 1 | N | 155160 | DNA-directed RNA polymerase subunit beta' OS=Escherichia coli (strain K12 / DH10B) OX=316385 GN=rpoC PE=3 SV=1 |
| 386 | 591 | P75914\|YCDX_ECOLI | 23,12 | 3 | 1 | 1 | N | 26890 | Probable phosphatase YcdX OS=Escherichia coli (strain K12) OX=83333 GN=ycdX PE=1 SV=1 |
| 386 | 592 | C4ZRX5\|YCDX_ECOBW | 23,12 | 3 | 1 | 1 | N | 26890 | Probable phosphatase YcdX OS=Escherichia coli (strain K12 / MC4100 / BW2952) OX=595496 GN=ycdX PE=3 SV=1 |
| 386 | 593 | B1X9E8\|YCDX_ECODH | 23,12 | 3 | 1 | 1 | N | 26890 | Probable phosphatase YcdX OS=Escherichia coli (strain K12 / DH10B) OX=316385 GN=ycdX PE=3 SV=1 |
| 237 | 393 | P31449\|YIDL_ECOLI | 23,08 | 4 | 1 | 1 | N | 33958 | Uncharacterized HTH-type transcriptional regulator YidL OS=Escherichia coli (strain K12) OX=83333 GN=yidL PE=4 SV=2 |
| 200 | 290 | P31474\|HSRA_ECOLI | 22,88 | 4 | 1 | 1 | Y | 51462 | Probable transport protein HsrA OS=Escherichia coli (strain K12) OX=83333 GN=hsrA PE=1 SV=1 |
| 236 | 384 | P77324\|PAOB_ECOLI | 22,84 | 4 | 1 | 1 | N | 33858 | Aldehyde oxidoreductase FAD-binding subunit PaoB OS=Escherichia coli (strain K12) OX=83333 GN=paoB PE=1 SV=1 |
| 381 | 581 | P76157\|YNFN_ECOLI | 22,76 | 51 | 1 | 1 | N | 5639 | Uncharacterized protein YnfN OS=Escherichia coli (strain K12) OX=83333 GN=ynfN PE=2 SV=2 |
| 288 | 583 | P37353\|MENE_ECOLI | 22,7 | 3 | 1 | 1 | N | 50185 | 2-succinylbenzoate--CoA ligase OS=Escherichia coli (strain K12) OX=83333 GN=menE PE=1 SV=2 |
| 232 | 320 | P45758\|GSPD_ECOLI | 22,62 | 1 | 1 | 1 | N | 70698 | Putative secretin GspD OS=Escherichia coli (strain K12) OX=83333 GN=gspD PE=1 SV=2 |
| 382 | 585 | P33934\|NAPH_ECOLI | 22,59 | 3 | 1 | 1 | N | 31874 | Ferredoxin-type protein NapH OS=Escherichia coli (strain K12) OX=83333 GN=napH PE=1 SV=1 |
| 384 | 587 | P16678\|PHNK_ECOLI | 22,43 | 8 | 1 | 1 | N | 27831 | Putative phosphonates utilization ATP-binding protein PhnK OS=Escherichia coli (strain K12) OX=83333 GN=phnK PE=1 SV=1 |
| 389 | 717 | P46481\|AAEB_ECOLI | 22,22 | 2 | 1 | 1 | N | 73591 | p-hydroxybenzoic acid efflux pump subunit AaeB OS=Escherichia coli (strain K12) OX=83333 GN=aaeB PE=1 SV=1 |
| 389 | 718 | C4ZSX8\|AAEB_ECOBW | 22,22 | 2 | 1 | 1 | N | 73588 | p-hydroxybenzoic acid efflux pump subunit AaeB OS=Escherichia coli (strain K12 / MC4100 / BW2952) OX=595496 GN=aaeB PE=3 SV=1 |
| 392 | 723 | B1XBA8\|KATG_ECODH | 22,04 | 2 | 1 | 1 | N | 80024 | Catalase-peroxidase OS=Escherichia coli (strain K12 / DH10B) OX=316385 GN=katG PE=3 SV=1 |
| 392 | 724 | P13029\|KATG_ECOLI | 22,04 | 2 | 1 | 1 | N | 80024 | Catalase-peroxidase OS=Escherichia coli (strain K12) OX=83333 GN=katG PE=1 SV=2 |
| 241 | 408 | P76585\|YPHG_ECOLI | 21,87 | 1 | 1 | 1 | N | 123832 | Uncharacterized protein YphG OS=Escherichia coli (strain K12) OX=83333 GN=yphG PE=4 SV=2 |
| 203 | 303 | P08331\|CPDB_ECOLI | 21,87 | 2 | 1 | 1 | N | 70832 | 2'_3'-cyclic-nucleotide 2'-phosphodiesterase/3'-nucleotidase OS=Escherichia coli (strain K12) OX=83333 GN=cpdB PE=1 SV=2 |
| 393 | 729 | P0CF91\|INSL1_ECOLI | 21,84 | 5 | 1 | 1 | N | 40909 | Putative transposase InsL for insertion sequence element IS186A OS=Escherichia coli (strain K12) OX=83333 GN=insL1 PE=3 SV=1 |
| 393 | 730 | P0CF92\|INSL2_ECOLI | 21,84 | 5 | 1 | 1 | N | 40909 | Putative transposase InsL for insertion sequence element IS186B OS=Escherichia coli (strain K12) OX=83333 GN=insL2 PE=3 SV=1 |
| 393 | 731 | P0CF93\|INSL3_ECOLI | 21,84 | 5 | 1 | 1 | N | 40909 | Putative transposase InsL for insertion sequence element IS186C OS=Escherichia coli (strain K12) OX=83333 GN=insL3 PE=3 SV=1 |
| 327 | 791 | B1XD46\|BAMA_ECODH | 21,75 | 1 | 1 | 1 | N | 90553 | Outer membrane protein assembly factor BamA OS=Escherichia coli (strain K12 / DH10B) OX=316385 GN=bamA PE=3 SV=1 |
| 327 | 792 | P0A940\|BAMA_ECOLI | 21,75 | 1 | 1 | 1 | N | 90553 | Outer membrane protein assembly factor BamA OS=Escherichia coli (strain K12) OX=83333 GN=bamA PE=1 SV=1 |
| 327 | 793 | C4ZRR9\|BAMA_ECOBW | 21,75 | 1 | 1 | 1 | N | 90553 | Outer membrane protein assembly factor BamA OS=Escherichia coli (strain K12 / MC4100 / BW2952) OX=595496 GN=bamA PE=3 SV=1 |
| 394 | 734 | P45763\|GSPL_ECOLI | 21,74 | 4 | 1 | 1 | N | 44540 | Putative type II secretion system protein L OS=Escherichia coli (strain K12) OX=83333 GN=gspL PE=2 SV=2 |
| 152 | 204 | tr\|A0A1V1IFN1\|A0A1V1IFN1_ECOLI | 21,68 | 5 | 1 | 1 | N | 48489 | Guanosine-inosine kinase OS=Escherichia coli (strain K12) OX=83333 GN=gsk-10 PE=3 SV=1 |
| 152 | 205 | tr\|A0A1V1IFM5\|A0A1V1IFM5_ECOLI | 21,68 | 5 | 1 | 1 | N | 48475 | Guanosine-inosine kinase OS=Escherichia coli (strain K12) OX=83333 GN=gsk-4 PE=3 SV=1 |
| 152 | 206 | tr\|A0A1V1IGN2\|A0A1V1IGN2_ECOLI | 21,68 | 5 | 1 | 1 | N | 48449 | Guanosine-inosine kinase OS=Escherichia coli (strain K12) OX=83333 GN=gsk PE=3 SV=1 |
| 152 | 207 | P0AEW6\|INGK_ECOLI | 21,68 | 5 | 1 | 1 | N | 48449 | Guanosine-inosine kinase OS=Escherichia coli (strain K12) OX=83333 GN=gsk PE=1 SV=1 |
| 152 | 208 | tr\|A0A1V1IGJ0\|A0A1V1IGJ0_ECOLI | 21,68 | 5 | 1 | 1 | N | 48521 | Guanosine-inosine kinase OS=Escherichia coli (strain K12) OX=83333 GN=gsk-1 PE=3 SV=1 |
| 152 | 209 | tr\|A0A1V1IGM6\|A0A1V1IGM6_ECOLI | 21,68 | 5 | 1 | 1 | N | 48491 | Guanosine-inosine kinase OS=Escherichia coli (strain K12) OX=83333 GN=gsk PE=3 SV=1 |
| 152 | 210 | tr\|A0A1V1IFN2\|A0A1V1IFN2_ECOLI | 21,68 | 5 | 1 | 1 | N | 48507 | Guanosine-inosine kinase OS=Escherichia coli (strain K12) OX=83333 GN=gsk-3 PE=3 SV=1 |
| 238 | 398 | P0AGA6\|UHPA_ECOLI | 21,54 | 10 | 1 | 1 | N | 20889 | Transcriptional regulatory protein UhpA OS=Escherichia coli (strain K12) OX=83333 GN=uhpA PE=1 SV=1 |
| 325 | 743 | P0AC41\|SDHA_ECOLI | 21,52 | 4 | 1 | 1 | Y | 64422 | Succinate dehydrogenase flavoprotein subunit OS=Escherichia coli (strain K12) OX=83333 GN=sdhA PE=1 SV=1 |
| 86 | 749 | P17117\|NFSA_ECOLI | 21,22 | 6 | 1 | 1 | Y | 26801 | Oxygen-insensitive NADPH nitroreductase OS=Escherichia coli (strain K12) OX=83333 GN=nfsA PE=1 SV=2 |
| 168 | 549 | P06715\|GSHR_ECOLI | 21,02 | 5 | 1 | 1 | Y | 48773 | Glutathione reductase OS=Escherichia coli (strain K12) OX=83333 GN=gor PE=1 SV=1 |
| 171 | 96 | P0A7A7\|PLSB_ECOLI | 20,98 | 1 | 1 | 1 | N | 91381 | Glycerol-3-phosphate acyltransferase OS=Escherichia coli (strain K12) OX=83333 GN=plsB PE=1 SV=2 |
| 171 | 97 | B1XC40\|PLSB_ECODH | 20,98 | 1 | 1 | 1 | N | 91381 | Glycerol-3-phosphate acyltransferase OS=Escherichia coli (strain K12 / DH10B) OX=316385 GN=plsB PE=3 SV=1 |
| 171 | 98 | C5A134\|PLSB_ECOBW | 20,98 | 1 | 1 | 1 | N | 91381 | Glycerol-3-phosphate acyltransferase OS=Escherichia coli (strain K12 / MC4100 / BW2952) OX=595496 GN=plsB PE=3 SV=1 |
| 171 | 99 | tr\|D0VE88\|D0VE88_ECOLI | 20,98 | 1 | 1 | 1 | N | 93584 | Glycerol-3-phosphate acyltransferase OS=Escherichia coli LW1655F+ OX=527799 GN=plsB PE=3 SV=1 |
| 119 | 485 | P0AFG3\|ODO1_ECOLI | 20,87 | 1 | 1 | 1 | N | 105062 | 2-oxoglutarate dehydrogenase E1 component OS=Escherichia coli (strain K12) OX=83333 GN=sucA PE=1 SV=1 |
| 405 | 767 | B1X716\|CYSG_ECODH | 20,76 | 2 | 1 | 1 | N | 49951 | Siroheme synthase OS=Escherichia coli (strain K12 / DH10B) OX=316385 GN=cysG PE=3 SV=1 |
| 405 | 768 | C4ZUM4\|CYSG_ECOBW | 20,76 | 2 | 1 | 1 | N | 49951 | Siroheme synthase OS=Escherichia coli (strain K12 / MC4100 / BW2952) OX=595496 GN=cysG PE=3 SV=1 |
| 405 | 769 | P0AEA8\|CYSG_ECOLI | 20,76 | 2 | 1 | 1 | N | 49951 | Siroheme synthase OS=Escherichia coli (strain K12) OX=83333 GN=cysG PE=1 SV=1 |
| 402 | 762 | P77713\|YAGH_ECOLI | 20,61 | 4 | 1 | 1 | Y | 60825 | Putative beta-xylosidase OS=Escherichia coli (strain K12) OX=83333 GN=yagH PE=3 SV=1 |
| 121 | 72 | P30958\|MFD_ECOLI | 20,51 | 2 | 1 | 1 | N | 129983 | Transcription-repair-coupling factor OS=Escherichia coli (strain K12) OX=83333 GN=mfd PE=1 SV=2 |
| 408 | 776 | C4ZZ41\|ILVD_ECOBW | 20,38 | 2 | 1 | 1 | N | 65532 | Dihydroxy-acid dehydratase OS=Escherichia coli (strain K12 / MC4100 / BW2952) OX=595496 GN=ilvD PE=3 SV=1 |
| 408 | 777 | P05791\|ILVD_ECOLI | 20,38 | 2 | 1 | 1 | N | 65532 | Dihydroxy-acid dehydratase OS=Escherichia coli (strain K12) OX=83333 GN=ilvD PE=1 SV=5 |
| 409 | 778 | B1XA16\|THIK_ECODH | 20,35 | 7 | 1 | 1 | Y | 32397 | Thiamine kinase OS=Escherichia coli (strain K12 / DH10B) OX=316385 GN=thiK PE=3 SV=1 |
| 409 | 779 | C4ZS46\|THIK_ECOBW | 20,35 | 7 | 1 | 1 | Y | 32397 | Thiamine kinase OS=Escherichia coli (strain K12 / MC4100 / BW2952) OX=595496 GN=thiK PE=3 SV=1 |
| 409 | 780 | P75948\|THIK_ECOLI | 20,35 | 7 | 1 | 1 | Y | 32397 | Thiamine kinase OS=Escherichia coli (strain K12) OX=83333 GN=thiK PE=1 SV=1 |
| 140 | 122 | P29018\|CYDD_ECOLI | 20,18 | 2 | 1 | 1 | N | 65056 | Glutathione/L-cysteine transport system ATP-binding/permease protein CydD OS=Escherichia coli (strain K12) OX=83333 GN=cydD PE=1 SV=3 |
| 425 | 820 | P09053\|AVTA_ECOLI | 20,15 | 2 | 1 | 1 | N | 46711 | Valine--pyruvate aminotransferase OS=Escherichia coli (strain K12) OX=83333 GN=avtA PE=1 SV=3 |
| 412 | 787 | P0AA76\|DGOT_ECOLI | 20,15 | 2 | 1 | 1 | N | 47077 | D-galactonate transporter OS=Escherichia coli (strain K12) OX=83333 GN=dgoT PE=1 SV=1 |
| 416 | 794 | P76558\|MAO2_ECOLI | 20,04 | 3 | 1 | 1 | Y | 82417 | NADP-dependent malic enzyme OS=Escherichia coli (strain K12) OX=83333 GN=maeB PE=1 SV=1 |
