# Supplementary material for: Sequence and structure analyses of lytic polysaccharide monooxygenases mined from metagenomic DNA of humus samples around white-rot fungi in Cuc Phuong tropical forest, Vietnam
Source: PeerJ. 2024 Jun 24;12:e17553. doi: 10.7717/peerj.17553 (PMC11210479; doi:10.7717/peerj.17553)
Supplement: Table S3 [file peerj-12-17553-s004.docx]

**Table S3. Taxonomic classification of 31 putative LPMOs mined form metagenomic DNA data of humus samples collected around white-rot fungi in primary forest Cuc Phuong, Vietnam**

| **Group** | **ID** | **Kingdom** | **Phyta** | **Class** | **Order** | **family** | **Genus** |
| --- | --- | --- | --- | --- | --- | --- | --- |
| 1 | **GL0125011** | Bacteria | Proteobacteria | Gammaproteobacteria | Enterobacterales |  |  |
|  | **GL0213284** | Bacteria | Proteobacteria | Gammaproteobacteria | Xanthomonadales | Xanthomonadaceae | Stenotrophomonas |
|  | **GL0247266** | Bacteria | Proteobacteria | Gammaproteobacteria | Enterobacterales | Enterobacteriaceae | Enterobacter |
|  | **GL0658692** | Bacteria | Proteobacteria | Gammaproteobacteria |  |  |  |
|  | **GL1034380** | Bacteria | Proteobacteria | Gammaproteobacteria | Pseudomonadales | Pseudomonadaceae | Pseudomonas |
| 2 | **GL0089352** | Bacteria | Proteobacteria | Gammaproteobacteria | Pseudomonadales | Pseudomonadaceae |  |
|  | **GL0393374** | Bacteria | Proteobacteria | Gammaproteobacteria | Pseudomonadales | Pseudomonadaceae |  |
|  | **GL0681738** | Bacteria | Proteobacteria | Gammaproteobacteria | Pseudomonadales | Pseudomonadaceae |  |
| 3.1 | **GL0183513** | Bacteria | Proteobacteria | Gammaproteobacteria | Pseudomonadales | Pseudomonadaceae | Pseudomonas |
|  | **GL0251010** | Bacteria | Proteobacteria | Gammaproteobacteria | Pseudomonadales | Pseudomonadaceae | Pseudomonas |
|  | **GL0422153** | Bacteria | Proteobacteria | Gammaproteobacteria | Pseudomonadales | Pseudomonadaceae |  |
|  | **GL0522565** | Bacteria | Proteobacteria | Gammaproteobacteria | Pseudomonadales | Pseudomonadaceae | Pseudomonas |
| 3.2 | **GL1004230** | Bacteria | Proteobacteria | Betaproteobacteria | Burkholderiales | Burkholderiaceae |  |
|  | **GL0293304** | Bacteria | Proteobacteria | Gammaproteobacteria | Enterobacterales | Yersiniaceae | Yersinia |
|  | **GL0883009** | Bacteria | Proteobacteria | Gammaproteobacteria | Pseudomonadales | Pseudomonadaceae |  |
| 3.3 | **GL0600730** | Bacteria | Proteobacteria | Gammaproteobacteria |  |  |  |
|  | **GL0200824** | Bacteria | Bacteroidetes | Flavobacteriia | Flavobacteriales | Flavobacteriaceae | Chryseobacterium |
|  | **GL0225724** | Bacteria | Firmicutes | Bacilli | Lactobacillales | Streptococcaceae | Lactococcus |
|  | **GL0338092** | Bacteria | Bacteroidetes | Flavobacteriia | Flavobacteriales | Flavobacteriaceae | Chryseobacterium |
|  | **GL0391320** | Bacteria | Bacteroidetes | Flavobacteriia | Flavobacteriales | Flavobacteriaceae | Chryseobacterium |
|  | **GL0656018** | Bacteria | Firmicutes | Bacilli | Bacillales | Bacillaceae |  |
| 3.4 | **GL0066553** | Bacteria | Proteobacteria | Gammaproteobacteria | Alteromonadales | Shewanellaceae | Shewanella |
|  | **GL0555809** | Bacteria | Proteobacteria | Gammaproteobacteria | Aeromonadales | Aeromonadaceae | Aeromonas |
|  | **GL0999597** | Bacteria | Proteobacteria | Gammaproteobacteria | Enterobacterales | Enterobacteriaceae |  |
| 3.5 | **GL0620585** | Bacteria | Proteobacteria | Gammaproteobacteria | Enterobacterales |  |  |
| 3.6 | **GL0297948** | Bacteria | Proteobacteria | Gammaproteobacteria | Enterobacterales | Enterobacteriaceae | Klebsiella |
|  | **GL0417116** | Bacteria | Proteobacteria | Gammaproteobacteria | Enterobacterales | Enterobacteriaceae |  |
|  | **GL0489328** | Bacteria | Proteobacteria | Gammaproteobacteria | Enterobacterales | Enterobacteriaceae |  |
|  | **GL0507050** | Bacteria | Proteobacteria | Gammaproteobacteria | Enterobacterales | Enterobacteriaceae | Enterobacter |
|  | **GL0772141** | Bacteria | Proteobacteria | Gammaproteobacteria | Enterobacterales | Enterobacteriaceae | Enterobacter |
|  | **GL0875000** | Bacteria | Proteobacteria | Gammaproteobacteria | Enterobacterales | Enterobacteriaceae | Enterobacter |
