# Supplementary material for: Sequence and structure analyses of lytic polysaccharide monooxygenases mined from metagenomic DNA of humus samples around white-rot fungi in Cuc Phuong tropical forest, Vietnam
Source: PeerJ. 2024 Jun 24;12:e17553. doi: 10.7717/peerj.17553 (PMC11210479; doi:10.7717/peerj.17553)
Supplement: Article S1 [file peerj-12-17553-s005.pdf]

Supplemental Article S1. Comparison of 31 putative LPMOs

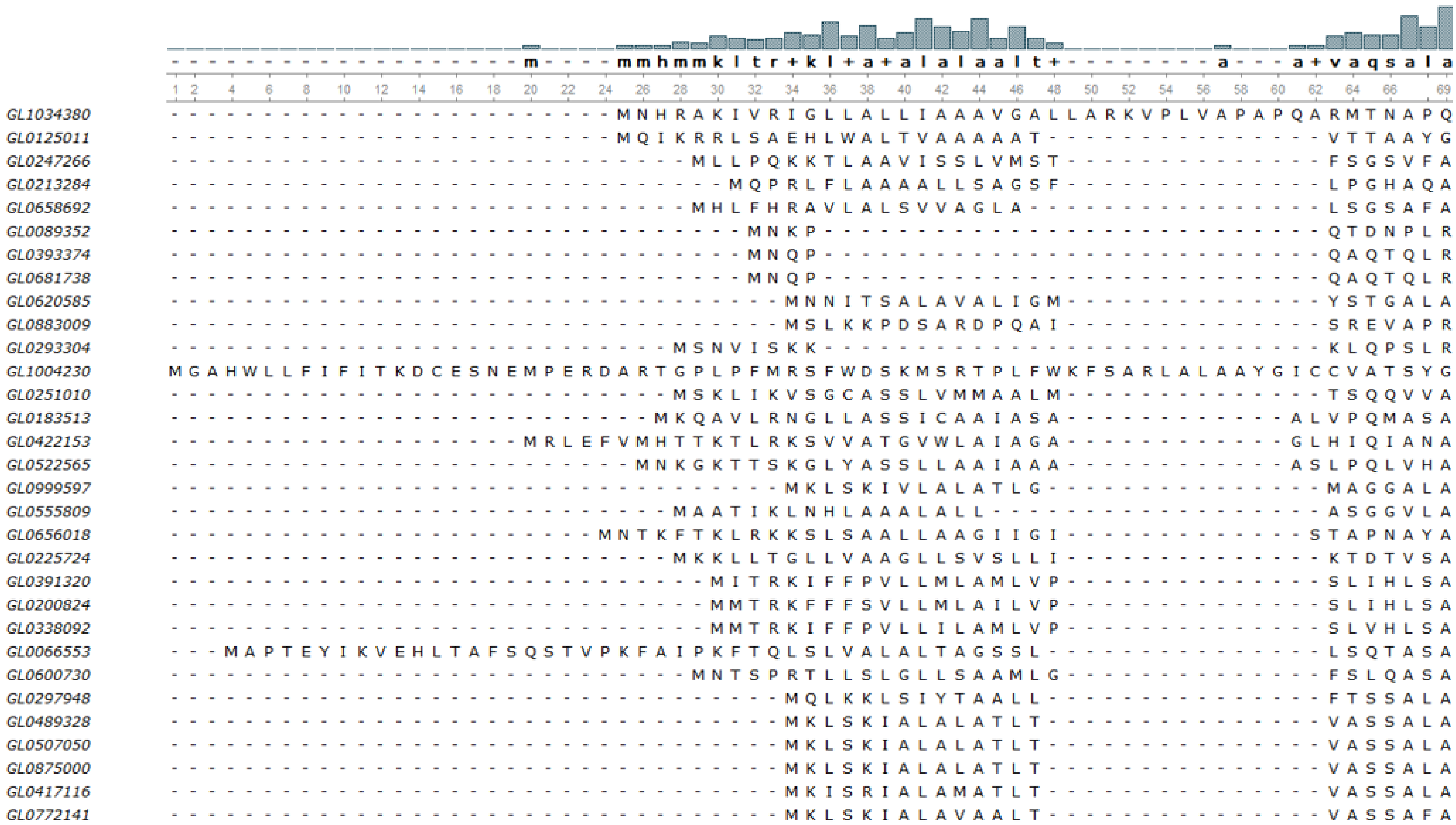

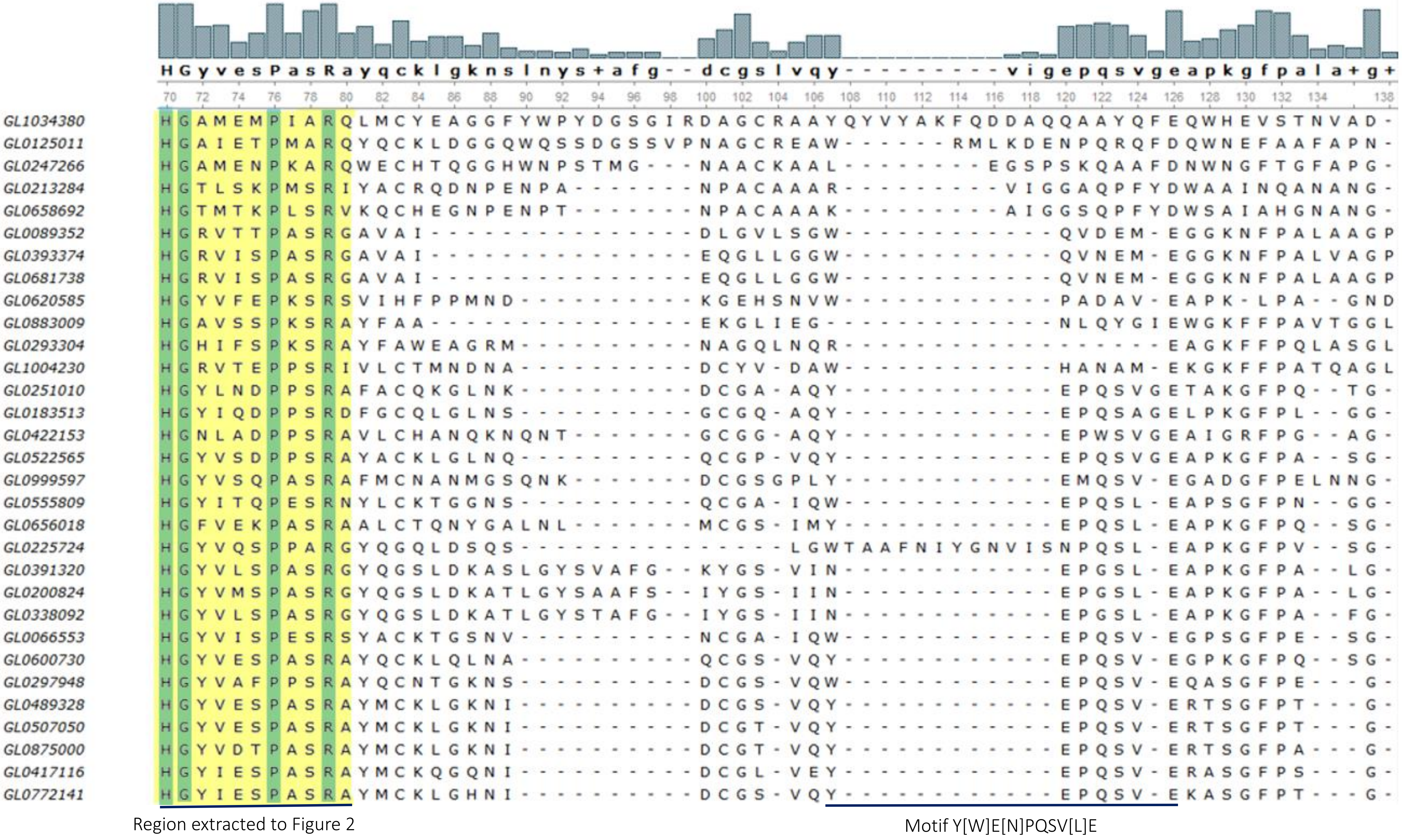
Region extracted to Figure 2
Motif Y[W]E[N]PQSV[L]E

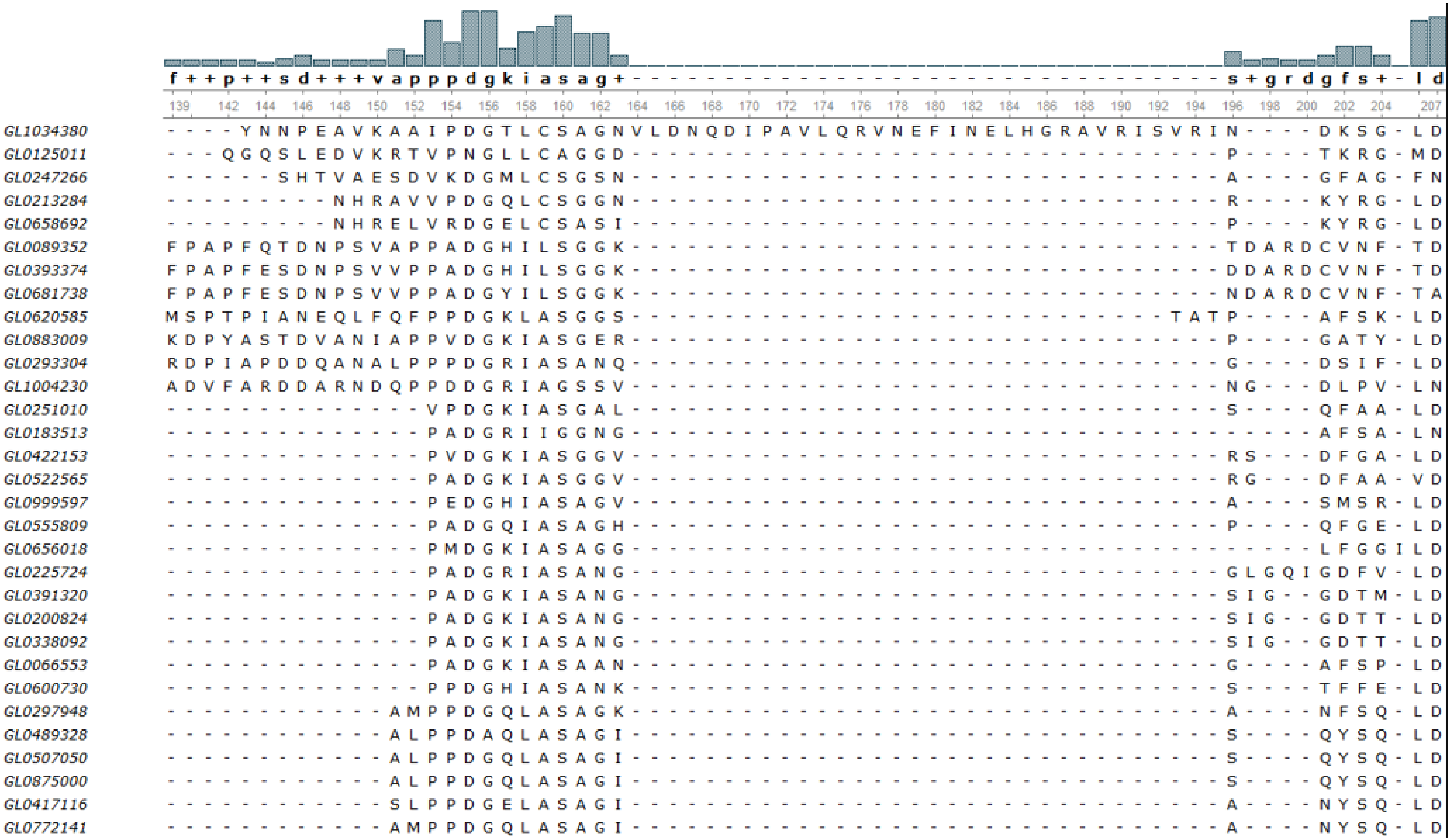
f++p++sd+++vapppdgkiasag+--------------------------------s+grdgfs+-ld
139 142 144 146 148 150 152 154 156 158 160 162 164 166 168 170 172 174 176 178 180 182 184 186 188 190 192 194 196 198 200 202 204 207
GL1034380 ----YNNPEAVKAAIPDGTLCSAGNVLDNQDIPAVLQRVNEFINELHGRAVRISVRIN----DKSG-LD
GL0125011 ---QGQSLEDVKRTVPNGLLCAGGD--------------------------------P----TKRG-MD
GL0247266 ------SHTVAESDVKDGMLCSGSN--------------------------------A----GFAG-FN
GL0213284 ---------NHRAVVPDGQLCSGGN--------------------------------R----KYRG-LD
GL0658692 ---------NHRELVRDGELCSASI--------------------------------P----KYRG-LD
GL0089352 FPAPFQTDNPSVAPPADGHILSGGK--------------------------------TDARDCVNF-TD
GL0393374 FPAPFESDNPSVVPPADGHILSGGK--------------------------------DDARDCVNF-TD
GL0681738 FPAPFESDNPSVVPPADGYILSGGK--------------------------------NDARDCVNF-TA
GL0620585 MSPTPIANEQLFQFPPDGKLASGGS-----------------------------TATP----AFSK-LD
GL0883009 KDPYASTDVANIAPPVDGKIASGER--------------------------------P----GATY-LD
GL0293304 RDPIAPDDQANALPPPDGRIASANQ--------------------------------G----DSIF-LD
GL1004230 ADVFARDDARNDQPPDDGRIAGSSV--------------------------------NG---DLPV-LN
GL0251010 --------------VPDGKIASGAL--------------------------------S----QFAA-LD
GL0183513 --------------PADGRIIGGNG---------------------------------------AFSA-LN
GL0422153 --------------PVDGKIASGGV--------------------------------RS---DFGA-LD
GL0522565 --------------PADGKIASGGV--------------------------------RG---DFAA-VD
GL0999597 --------------PEDGHIASAGV--------------------------------A----SMSR-LD
GL0555809 --------------PADGQIASAGH--------------------------------P----QFGE-LD
GL0656018 --------------PMDGKIASAGG---------------------------------------LFGGILD
GL0225724 --------------PADGRIASANG--------------------------------GLGQIGDFV-LD
GL0391320 --------------PADGKIASANG--------------------------------SIG--GDTM-LD
GL0200824 --------------PADGKIASANG--------------------------------SIG--GDTT-LD
GL0338092 --------------PADGKIASANG--------------------------------SIG--GDTT-LD
GL0066553 --------------PADGKIASAAN--------------------------------G----AFSP-LD
GL0600730 --------------PPDGHIASANK--------------------------------S----TFFE-LD
GL0297948 ------------AMPPDGQLASAGK--------------------------------A----NFSQ-LD
GL0489328 ------------ALPPDAQLASAGI--------------------------------S----QYSQ-LD
GL0507050 ------------ALPPDGQLASAGI--------------------------------S----QYSQ-LD
GL0875000 ------------ALPPDGQLASAGI--------------------------------S----QYSQ-LD
GL0417116 ------------SLPPDGELASAGI--------------------------------A----NYSQ-LD
GL0772141 ------------AMPPDGQLASAGI--------------------------------A----NYSQ-LD

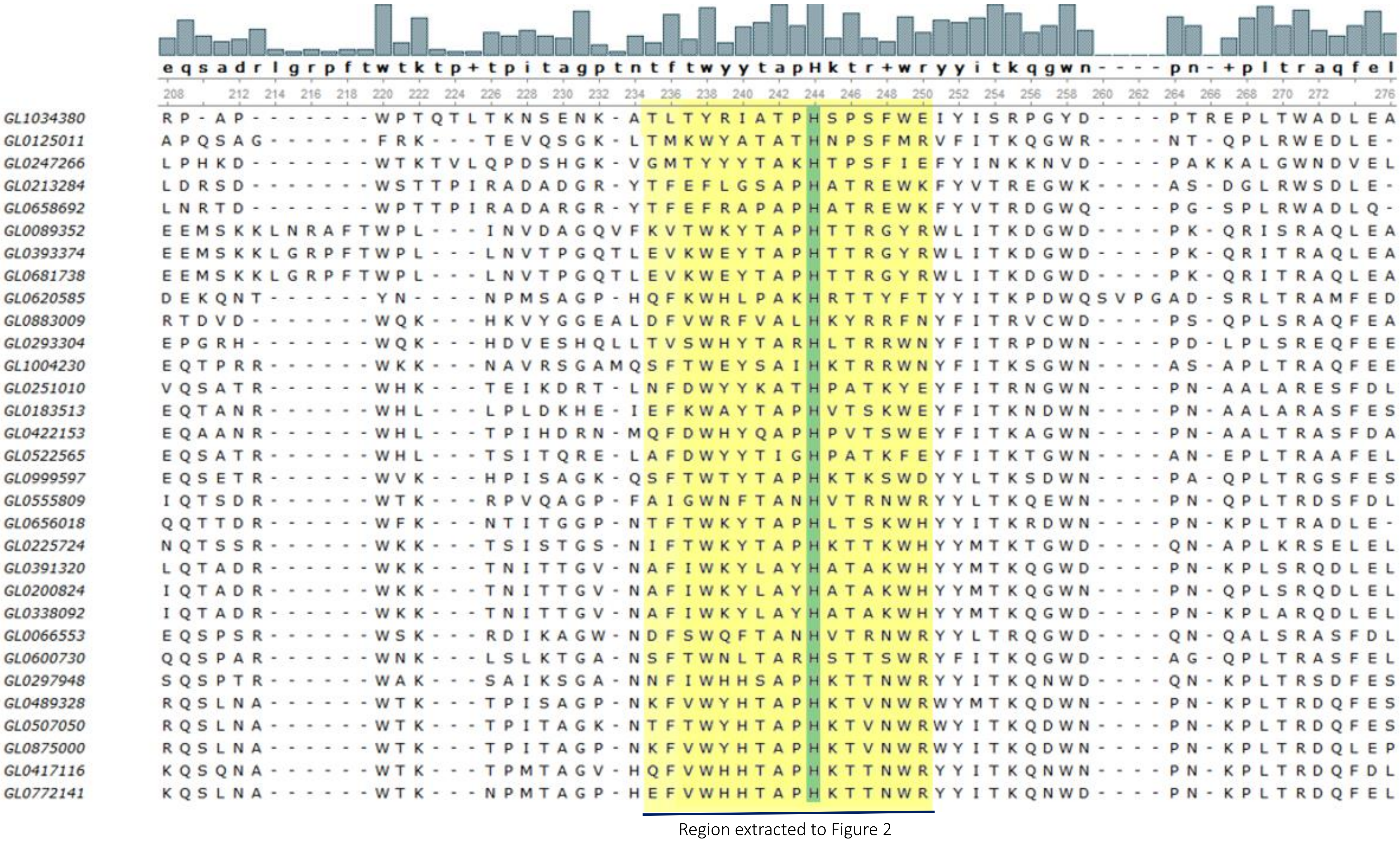
eqsadrlgrpftwtktp+tpitagptntftwyytapHktr+wryyitkqgwn----pn-+pltraqfel
GL1034380
GL0125011
GL0247266
GL0213284
GL0658692
GL0089352
GL0393374
GL0681738
GL0620585
GL0883009
GL0293304
GL1004230
GL0251010
GL0183513
GL0422153
GL0522565
GL0999597
GL0555809
GL0656018
GL0225724
GL0391320
GL0200824
GL0338092
GL0066553
GL0600730
GL0297948
GL0489328
GL0507050
GL0875000
GL0417116
GL0772141
Region extracted to Figure 2

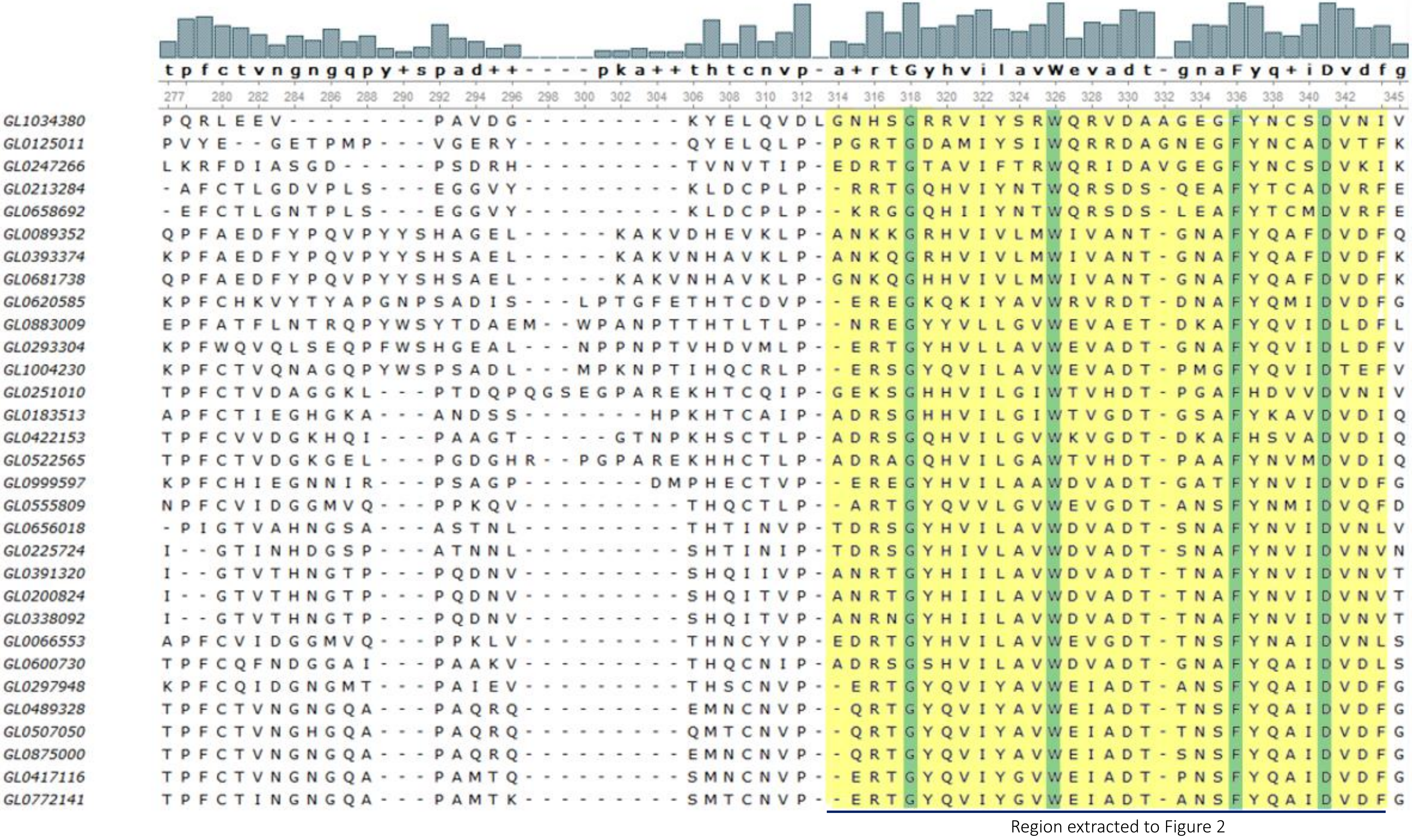
tpfctvngngqpy+spad++----pka++thtcnvp-a+rtGyhvilavWevadt-gnaFyq+iDvdfg
277 280 282 284 286 288 290 292 294 296 298 300 302 304 306 308 310 312 314 316 318 320 322 324 326 328 330 332 334 336 338 340 342 345
GL1034380 PQRLEEV--------PAVDG---------KYELQVDLGNHSGRRVIYSRWQRVDAAGEGFYNCSDVNIV
GL0125011 PVYE--GETPMP---VGERY---------QYELQLP-PGRTGDAMIYSIWQRRDAGNEGFYNCADVTFK
GL0247266 LKRFDIASGD-----PSDRH---------TVNVTIP-EDRTGTAVIFTRWQRIDAVGEGFYNCSDVKIK
GL0213284 -AFCTLGDVPLS---EGGVY---------KLDCPLP--RRTGQHVIYNTWQRSDS-QEAFYTCADVRFE
GL0658692 -EFCTLGNTPLS---EGGVY---------KLDCPLP--KRGGQHIIYNTWQRSDS-LEAFYTCMDVRFE
GL0089352 QPFAEDFYPQVPYYSHAGEL-----KAKVDHEVKLP-ANKKGRHVIVLMWIVANT-GNAFYQAFDVDFQ
GL0393374 KPFAEDFYPQVPYYSHSAEL-----KAKVNHAVKLP-ANKQGRHVIVLMWIVANT-GNAFYQAFDVDFK
GL0681738 QPFAEDFYPQVPYYSHSAEL-----KAKVNHAVKLP-GNKQGHHVIVLMWIVANT-GNAFYQAFDVDFK
GL0620585 KPFCHKVYTYAPGNPSADIS---LPTGFETHTCDVP--EREGKQKIYAVWRVRDT-DNAFYQMIDVDFG
GL0883009 EPFATFLNTRQPYWSYTDAEM--WPANPTTHTLTLP--NREGYYVLLGVWEVAET-DKAFYQVIDLDFL
GL0293304 KPFWQVQLSEQPFWSHGEAL---NPPNPTVHDVMLP--ERTGYHVLLAVWEVADT-GNAFYQVIDLDFV
GL1004230 KPFCTVQNAGQPYWSPSADL---MPKNPTIHQCRLP--ERSGYQVILAVWEVADT-PMGFYQVIDTEFV
GL0251010 TPFCTVDAGGKL---PTDQPQGSEGPAREKHTCQIP-GEKSGHHVILGIWTVHDT-PGAFHDVVDVNIV
GL0183513 APFCTIEGHGKA---ANDSS-------HPKHTCAIP-ADRSGHHVILGIWTVGDT-GSAFYKAVDVDIQ
GL0422153 TPFCVVDGKHQI---PAAGT-----GTNPKHSCTLP-ADRSGQHVILGVWKVGDT-DKAFHSVADVDIQ
GL0522565 TPFCTVDGKGEL---PGDGHR--PGPAREKHHCTLP-ADRAGQHVILGAWTVHDT-PAAFYNVMDVDIQ
GL0999597 KPFCHIEGNNIR---PSAGP-------DMPHECTVP--EREGYHVILAAWDVADT-GATFYNVIDVDFG
GL0555809 NPFCVIDGGMVQ---PPKQV---------THQCTLP--ARTGYQVVLGVWEVGDT-ANSFYNMIDVQFD
GL0656018 -PIGTVAHNGSA---ASTNL---------THTINVP-TDRSGYHVILAVWDVADT-SNAFYNVIDVNLV
GL0225724 I--GTINHDGSP---ATNNL---------SHTINIP-TDRSGYHIVLAVWDVADT-SNAFYNVIDVNVN
GL0391320 I--GTVTHNGTP---PQDNV---------SHQIIVP-ANRTGYHIILAVWDVADT-TNAFYNVIDVNVT
GL0200824 I--GTVTHNGTP---PQDNV---------SHQITVP-ANRTGYHIILAVWDVADT-TNAFYNVIDVNVT
GL0338092 I--GTVTHNGTP---PQDNV---------SHQITVP-ANRNGYHIILAVWDVADT-TNAFYNVIDVNVT
GL0066553 APFCVIDGGMVQ---PPKLV---------THNCYVP-EDRTGYHVILAVWEVGDT-TNSFYNAIDVNLS
GL0600730 TPFCQFNDGGAI---PAAKV---------THQCNIP-ADRSGSHVILAVWDVADT-GNAFYQAIDVDLS
GL0297948 KPFCQIDGNGMT---PAIEV---------THSCNVP--ERTGYQVIYAVWEIADT-ANSFYQAIDVDFG
GL0489328 TPFCTVNGNGQA---PAQRQ---------EMNCNVP--QRTGYQVIYAVWEIADT-TNSFYQAIDVDFG
GL0507050 TPFCTVNGHGQA---PAQRQ---------QMTCNVP--QRTGYQVIYAVWEIADT-TNSFYQAIDVDFG
GL0875000 TPFCTVNGNGQA---PAQRQ---------EMNCNVP--QRTGYQVIYAVWEIADT-SNSFYQAIDVDFG
GL0417116 TPFCTVNGNGQA---PAMTQ---------SMNCNVP--ERTGYQVIYGVWEIADT-PNSFYQAIDVDFG
GL0772141 TPFCTINGNGQA---PAMTK---------SMTCNVP--ERTGYQVIYGVWEIADT-ANSFYQAIDVDFG
Region extracted to Figure 2

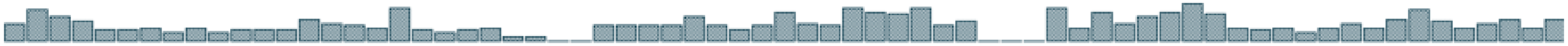

```
           gggg vtpvdatptppsewqktlls--gsitagndln+gdkvia---r+fdangevaslvteltiasagq
           346 350 352 354 356 358 360 362 364 366 368 370 372 374 376 378 380 382 384 386 388 390 392 394 396 398 400 402 404 406 408 410 414
GL1034380  AGP------------------------------------------------------------------
GL0125011  GNGT--------TPPSPWHYKG----PFVTPSLSPVAGDKVRF---RVLTGPGKQEIVDERIDVTAQNL
GL0247266  SRDGSEIPDGGGEEEGGDESDWISKGDFITAAHQPAVGEQVRF---RLMGGTRGDNLVDVYLKITAQNI
GL0213284  GGDV--------TPPPQWQDA-----GALTARSALEVGTTVSL---RVFNVAGNDVERVD--VVLASGQ
GL0658692  GGDG-------VVPPPQWQDA-----GAVTANGALDVDSTVTL---RVFNANGNDLERVE--TTLAAGQ
GL0089352  ---------------------------------------------------------------------
GL0393374  ---------------------------------------------------------------------
GL0681738  ---------------------------------------------------------------------
GL0620585  GEVITKPVANVTPSSHNLSTSTLSLDGSASTGKELKYSWAVTSNADKVILENSTSAKASIRLKAKPE--
GL0883009  PGGEGP---------------------------------------------------------------
GL0293304  GASV---------EPLPAAPAGLRATSVSTGTVALAWNAPSIPASSYRVYRDNVLITAVSGLTFNDEGL
GL1004230  SGNM-----------------------------------------------------------------
GL0251010  AEAE---------NPDGWRPV-----GNITPHADLWVGDKVKV---RALTADGESAAYSSQLTIASQED
GL0183513  ADGG---------PAPEWRQV-----SSINPHRDLKVGDEVKA---RAFIGGNESQEFSASISIDTVEA
GL0422153  LDGG---------PAPEWPRL-----AEISANRDLEVGDKVTA---RAFDANGERTSHHVSITVANAEE
GL0522565  IEGG---------TPPQWPQV-----GSITPHRDLRQGDKVKA---RAFIGTTESTQHSVSISIDNAED
GL0999597  G--------------------------------------------------------------------
GL0555809  GGTQ---------PPLTWSQG-----GTIYPSIDLVAGDKART---RVFDANGERTDLQTVLTIGSAEQ
GL0656018  NNDGNVDTEAPTQPNGLHTTKV----TSTSAELKWNPSTDNVGVKEYQVLRDGKVVATIPGTTFTDKNL
GL0225724  NKNS-----------------------------------------------------------------
GL0391320  SGTG---VSAPATPTGLTQIGV----TSSSAKMSWIPQPDAVS---YAVFRNGQSIQQVSVAAFEDTGL
GL0200824  SGTG---VSAPATPTGLTQVGV----TSSSAKISWNAQTDAAS---YNVFRNGQNIQQVSTPTFQDTGL
GL0338092  SGTG---VAAPATPTGLAQVGV----TSSSAKISWNPQSDAVS---YSVFRNGQNIQQVSTPTFQDMGL
GL0066553  SGAV---------VPGEWTDI-----GDINPSLDLKAGDKVMT---RVFDANGEQSAKQTQITIADATQ
GL0600730  K--------------------------------------------------------------------
GL0297948  GTGD------DAENGSLWTTQL----TGQLSGKDLHAGDKVIA---HFFNASGEVHSLQTELTIASEAQ
GL0489328  NAGNVTPSDDTPVVVSEWNKTL----DGQIAGNNVNEGDKIIA---RFFDANGEVTSLRTEMTVASASQ
GL0507050  NGGNVTP-DETPAVVSEWTNTL----NGQISGNNVNAGDKVIA---RFFDANGEVVSMRTELTVSSASQ
GL0875000  NGGNVTP-DETPAVVSEWNKTL----DGQISGNNVNAGDKVIA---RFFDANGEVASMRTELTVSSASQ
GL0417116  NGGNVTP-DETPAAASVWSKTL----SGQVAGNNLNVGDKVIA---RFFDANGEIASMATELTIASAQQ
GL0772141  NGGNVTP-DDTPAVISQWSKTL----SGQVAGNNLNTGDKVIA---RFFDANGEVAALRTELNIGSAAQ
```

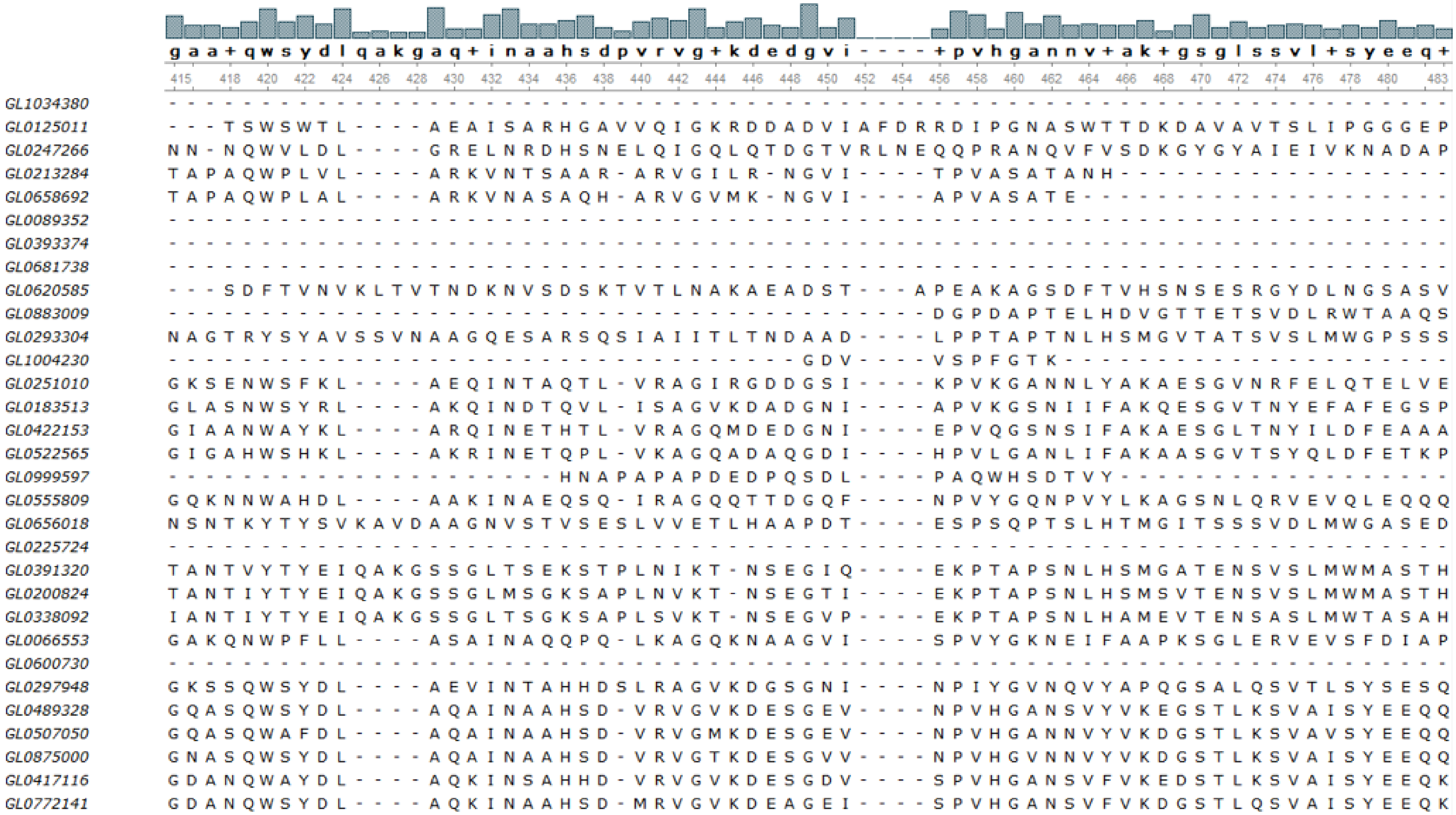
g a a + q w s y d l q a k g a q + i n a a h s d p v r v g + k d e d g v i - - - - + p v h g a n n v + a k + g s g l s s v l + s y e e q +
415 418 420 422 424 426 428 430 432 434 436 438 440 442 444 446 448 450 452 454 456 458 460 462 464 466 468 470 472 474 476 478 480 483
GL1034380 ---
GL0125011 ---TSWSWTL----AEAISARHGAVVQIGKRDDADVIAFDRRDIPGNASWTTDKDAVAVTSLIPGGGEP
GL0247266 NN-NQWVLDL----GRELNRDHSNELQIGQLQTDGTVRLNEQQPRANQVFVSDKGYGYAIEIVKNADAP
GL0213284 TAPAQWPLVL----ARKVNTSAAR-ARVGILR-NGVI----TPVASATANH------------------
GL0658692 TAPAQWPLAL----ARKVNASAQH-ARVGVMK-NGVI----APVASATE--------------------
GL0089352 ---
GL0393374 ---
GL0681738 ---
GL0620585 ---SDFTVNVKLTVTNDKNVSDSKTVTLNAKAEADST---APEAKAGSDFTVHSNSESRGYDLNGSASV
GL0883009 ---DGPDAPTELHDVGTTETSVDLRWTAAQS
GL0293304 NAGTRYSYAVSSVNAAGQESARSQSIAIITLTNDAAD----LPPTAPTNLHSMGVTATSVSLMWGPSSS
GL1004230 ----------------------------------GDV----VSPFGTK---------------------
GL0251010 GKSENWSFKL----AEQINTAQTL-VRAGIRGDDGSI----KPVKGANNLYAKAESGVNRFELQTELVE
GL0183513 GLASNWSYRL----AKQINDTQVL-ISAGVKDADGNI----APVKGSNIIFAKQESGVTNYEFAFEGSP
GL0422153 GIAANWAYKL----ARQINETHTL-VRAGQMDEDGNI----EPVQGSNSIFAKAESGLTNYILDFEAAA
GL0522565 GIGAHWSHKL----AKRINETQPL-VKAGQADAQGDI----HPVLGANLIFAKAASGVTSYQLDFETKP
GL0999597 ---------------------HNAPAPAPDEDPQSDL----PAQWHSDTVY------------------
GL0555809 GQKNNWAHDL----AAKINAEQSQ-IRAGQQTTDGQF----NPVYGQNPVYLKAGSNLQRVEVQLEQQQ
GL0656018 NSNTKYTYSVKAVDAAGNVSTVSESLVVETLHAAPDT----ESPSQPTSLHTMGITSSSVDLMWGASED
GL0225724 ---
GL0391320 TANTVYTYEIQAKGSSGLTSEKSTPLNIKT-NSEGIQ----EKPTAPSNLHSMGATENSVSLMWMASTH
GL0200824 TANTIYTYEIQAKGSSGLMSGKSAPLNVKT-NSEGTI----EKPTAPSNLHSMSVTENSVSLMWMASTH
GL0338092 IANTIYTYEIQAKGSSGLTSGKSAPLSVKT-NSEGVP----EKPTAPSNLHAMEVTENSASLMWTASAH
GL0066553 GAKQNWPFLL----ASAINAQQPQ-LKAGQKNAAGVI----SPVYGKNEIFAAPKSGLERVEVSFDIAP
GL0600730 ---
GL0297948 GKSSQWSYDL----AEVINTAHHDSLRAGVKDGSGNI----NPIYGVNQVYAPQGSALQSVTLSYSESQ
GL0489328 GQASQWSYDL----AQAINAAHSD-VRVGVKDESGEV----NPVHGANSVYVKEGSTLKSVAISYEEQQ
GL0507050 GQASQWAFDL----AQAINAAHSD-VRVGMKDESGEV----NPVHGANNVYVKDGSTLKSVAVSYEEQQ
GL0875000 GNASQWSYDL----AQAINAAHSD-VRVGTKDESGVV----NPVHGVNNVYVKDGSTLKSVAISYEEQQ
GL0417116 GDANQWAYDL----AQKINSAHHD-VRVGVKDESGDV----SPVHGANSVFVKEDSTLKSVAISYEEQK
GL0772141 GDANQWSYDL----AQKINAAHSD-MRVGVKDEAGEI----SPVHGANSVFVKDGSTLQSVAISYEEQK

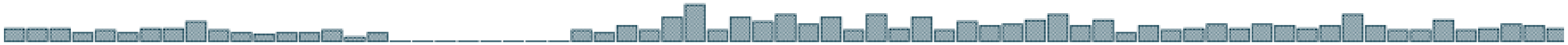

```
             aqv+e++avs++gfs+e--------yki+ngkatvtf+vtt+gdvnlta+vf+hyga++gyvkqq+nn+
             484 488 490 492 494 496 498 500 502 504 506 508 510 512 514 516 518 520 522 524 526 528 530 532 534 536 538 540 542 544 546 548 552
GL1034380    ---------------------------------------------------------------------
GL0125011    DPVAPPQAKISGPSTVK---------------AGETITLTNEGTNNTTGQHYRYDWHAHGFTPMNGTAS
GL0247266    AISLDRYTLAPIATTTSGYSYAVTGSSDKSGVTWQWKNVAGDGRITASPANQAKTQITVPGGVPAGTTA
GL0213284    --------------------------------------------VYLRPGHRFQLDTKVPDPVDPVDPP
GL0658692    ------------------------------------------NRVYLKPGNRFQLETQVPGPVDPVDPP
GL0089352    ---------------------------------------------------------------------
GL0393374    ---------------------------------------------------------------------
GL0681738    ---------------------------------------------------------------------
GL0620585    NAEKYQWTIVSGQDIGA--------LQVANGGEWVSTVHAAKARALIKPGKIGKVTYRLTVTAKNGKTS
GL0883009    GGPIDGFIIYRDG----------------NEVGRVPASQRTFTDDNLTPDTRYRYTVKAFDAQGQLSAS
GL0293304    VNALQGYIVYRDG----------------NEISRLPASQLSYLDTNVAPANSYRYFVAAIDVQGRLSVP
GL1004230    ---------------------------------------------------------------------
GL0251010    DS---GASLSIQAFDKT--------VELVKGRATLPLTVLGNRTMNIEATVFDASHKAVGSATRVMEPG
GL0183513    PD----AYMHLHDLKSE--------YVLKDGEATVGFSVMTNRSLEVTATLFDATNKQVGFQRQQVNST
GL0422153    GE---PTTMHLHDVKPE--------YTIVDGKGGVDFSVMTNKALTVTARLFDSNNKQVSYSRQNVN-G
GL0522565    VD---DAYLHLHGIKPE--------YVLKDGKGAVDFSVMTNRKLQVTSQLFDEHNQQVGHASQLVDAS
GL0999597    ---------------------------------------------------------------------
GL0555809    PPASDSISVS--GLESE--------YLLDNGKVTLAFTVTAEGDLAVTNTLYDHGGAAKGQTSADIKDS
GL0656018    NVAVDHYVIYRGSTKEQ-------------MTAVGTSTITSFKDMNLQSNTSYIYAVAAVDVAGNESIK
GL0225724    ---------------------------------------------------------------------
GL0391320    TQGIKNYQVF------------------ENGIKVGETVQTSFSRVGLAQDTEYLYTVRSVAMNDQISDM
GL0200824    SQGIKNYQIF------------------ENGIKVGETMQTNFLRTGLTQDTEYRYTVKSVAMNDQLSDA
GL0338092    SQGIKNYHVF------------------ENGIKVAETVQTTFLRTGLSQDTEYNYTVRSVGMNDQLSEV
GL0066553    AP---GNQLNVTSLADD--------YTIVDGVAQVSFDVSTNADMQVSAYLFSHDGTAAGYVTQAVNNT
GL0600730    ---------------------------------------------------------------------
GL0297948    EDDTAKESLSLSNVAAT---------PIENGKATVTFNAAAKGEMHLIAVVSDHAGAEKGSLQQTIDNR
GL0489328    AQVSEDIAVTNVQFS-----------KIKSGAATVTFHVNTQGNVNLEARVMNHAGAEKGYVKQEMNNA
GL0507050    SQVSEAIAVTNVKFS-----------KIKNGAATVTFHVNTQGNVNLEARVMNHAGAEKGYVKQDMNNA
GL0875000    AQVSENIAVSNVKFS-----------KIANGNATVTFHVNTQGNVNLEARVMNHAGAEKGYLKQDMNNA
GL0417116    AVVNESIAVTDLHYS-----------KVTHGAATVTFHVNTQGDVNLEAHVLNHPGAEKGYLKQDMNNS
GL0772141    AEVHETIAVSNLQYS-----------NIENGKATVTFHVNTKGDVNVEAHVMSHSGAEKGYLKQEMNNA
```

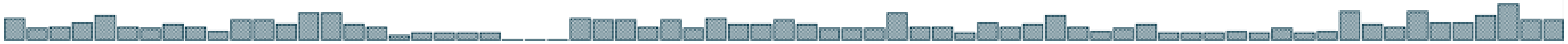

```
                s+dltvn+ksvtpghhmsycga---lkyvasnkdgtafaqdv+dlmlegna+yanpge+ggydyvfpeg
                553  556 558 560 562 564 566 568 570 572 574 576 578 580 582 584 586 588 590 592 594 596 598 600 602 604 606 608 610 612 614 616 618 621
GL1034380       ---------------------------------------------------------------------
GL0125011       SWSGTAPATPGKAT-----------ATLVVTDIEGRTSRATHDITITGEN--------GGGLHPAWPAG
GL0247266       TFELTGKTANGTPGKATLK------VTVQAPQVAPTGPTSIASDKGGKFTAKANFDYSQGNVSYSWSLL
GL0213284       ----------------------------------------------------------PGDYDYVYPAG
GL0658692       ----------------------------------------------------------GGDFDFVYPAG
GL0089352       ---------------------------------------------------------------------
GL0393374       ---------------------------------------------------------------------
GL0681738       ---------------------------------------------------------------------
GL0620585       TDDITVTVKEAQQASDVQINTGNNQDTIVGSDVIAAAVVSKNNDSLKGAKIEWSALNHADKIQISSPNN
GL0883009       SNVHDAHTKPK-----------------------------------------------EDGGDYRDWKL
GL0293304       GNVLTVTTLTQD----------------------------------------------NGGGDDNAEQG
GL1004230       ---------------------------------------------------------------------
GL0251010       SATLEIALLSEPGAHQ---------IKLIGSTTDGRTTRQRLESLELTGEGG------GQAYDAVYPEG
GL0183513       TSPFAVQASSVDGEHT---------LKIVGKNADEEILLQEDRPLKLMTQ--------DDAAYPTFPEG
GL0422153       TEPFALNVVSAEGEHT---------LKVVGVDKRERVLLQDEKTVQLKAAG-------DASHDFVFPES
GL0522565       TAPVVLEASSGAGKHL---------LKVVGVDNDKRVVLQEEREVRLLDAG-------NAEHDFVFPQS
GL0999597       ----------------------------------------------------------VGGDRVTW---
GL0555809       SHSFTIEVSGLSAGHHQ--------LVIEGKPKAGGAPLQQTLDLMFKDPAS------GGDYQYSFPEG
GL0656018       SSILTVTTK-------------------------------------------------EQSTSYEQWSP
GL0225724       ---------------------------------------------------------------------
GL0391320       SNELKVRTKKITPGNGQTYCGA---EQYNSANAYPTAGAKVFYACKIWKNKWYANPGELPGTNMVW-EE
GL0200824       SNELKIRTKKVTPGNGQSYCGA---EQYNAANAYPTAGVKVFYSCKIWKNKWYANPGELPGTNMVW-EE
GL0338092       SNTLKVRTKKVTPGNGQSYCGA---EPYNAANAYPIAGVKVFYACKIWKNKWYANPGEVPGTNMVW-EE
GL0066553       SASFVLDVVAPKAGHY---------HLQVKAEPKQGEVIQQNFDLFLKDQTT------APDADFIFPEG
GL0600730       ---------------------------------------------------------------------
GL0297948       SLPLSIPLTNVQAGHHM--------LRYSASNAQGAIISQGVINLMLEEKSEPTPAP-AGNYDYTFPDN
GL0489328       NQDITMNLSGVTAGHHM--------LKYYATNKDGTLFAQDVVDMMLENDASADTGT-TGKYDYTFPQS
GL0507050       NQDVTMNLSDVTPGHHM--------LKYYASNKDGVMFAQDVIDMMLEGEASNTDNGTAGNYEFIFPQN
GL0875000       NQDVTLNLTDVTPGHHM--------LKYYAVNKDGTLFAQDVVDMMLEGEAASGNTGNTGNFDYTFPEN
GL0417116       NQDVTMNLTNVTPGHHM--------LKYYATNKAGTLFAQDVLDMMLEGEATSGD---TGKSDFIFPDN
GL0772141       NQDVTMTLTDVTAGHHM--------LKYYASNKDGTLFDQDVLNLMLENNAADS----TGQHDVIFPDG
```

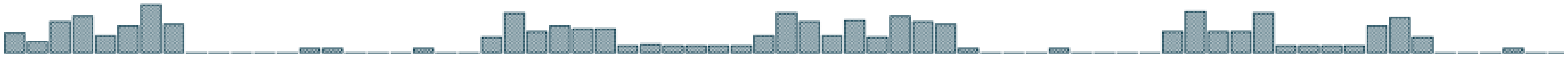

```
          i+sykagt-----nv---l--kvlqpkpvtycg+gkvyeckpa---v----fpysgtkvfycv---y--
          622 626 628 630 632 634 636 638 640 642 644 646 648 650 652 654 656 658 660 662 664 666 668 670 672 674 676 678 680 682 684 686 690
GL1034380 ---------------------------------------------------------------------
GL0125011 LGTYKPGD--------------VVTGL------DGRAWACKP---------FPHGG----WCN------
GL0247266 KGSSEVSGIDQSGNVKSGLAAGDYQVKVTAELDNGERKASGTAVLKVTDKDEPGTGDYESWVAGKIYY-
GL0213284 IGSYVPGQ-------------TVVKGN------DGKLYACRP---------FPEGA----WCN------
GL0658692 LGSYTPGE-------------TVVKGT------DGKLYACRP---------FPEGA----WCN------
GL0089352 ---------------------------------------------------------------------
GL0393374 ---------------------------------------------------------------------
GL0681738 ---------------------------------------------------------------------
GL0620585 FSTNLMGKHPYTDNVDVTLQVKVTAPDTGKVSTATKVIKLRPAQTDVKA--WDHSKTYKTPCEKVSYEG
GL0883009 KDSYTTGE-------------VVRH--------NQTLWQCLA---------------------------
GL0293304 LRQWVRGT-------------TWYTGERVRH--NGLIWVCLQ-------------------QH------
GL1004230 ---------------------------------------------------------------------
GL0251010 IDSYVAGT-------------TVLQPE------DGKVYECKP---------FPAAG----WCK------
GL0183513 LAGYTAGT-------------KVYAKG------TTDVYECKP---------FPYSG----YCV------
GL0422153 LSSYKAGT-------------KVLQPK------NGKVYECQP---------FPNSG----YCV------
GL0522565 IETYRAGT-------------LVLQGK------TGEVFECKP---------FPAEG----WCR------
GL0999597 ---------------------------------NGKEYQAR----------------------------
GL0555809 LKSYAAGT-------------KVLQPK------DGRIYQCKP---------YPYSG----WCT------
GL0656018 FKAYKKGD-------------KVEY--------QGKNYEAVQ----------SYQG----YGD------
GL0225724 ---------------------------------SSQVFGPFL---------------------------
GL0391320 ISVCTEGP-------------GCQSSGPVTYCGAQEYNPAKT---------YPTAGTKVFYAC------
GL0200824 VSTCTEGP-------------GCESSGPVTYCGAQEYNPAKT---------YPTAGVKVFYSF------
GL0338092 VSACTEGP-------------GCESSGPVTYCGAQEYNPAKA---------YPTAGTKVFHAC------
GL0066553 IKSYAAGT-------------KVLQPK------TGKVYQCKP---------WPYSG----YCV------
GL0600730 ---------------------------------------------------------------------
GL0297948 LKSYTAGT-------------KVMQPK------NGKVYQCRP---------FPYSG----YCV------
GL0489328 IASYKAGT-------------VVLQPK------NGKTYQCKK---------APYNG----YCV------
GL0507050 IASYKAGT-------------VVLQPK------NGKTYKCKT---------APYSG----YCI------
GL0875000 IKSYKAGT-------------VVLQPK------DGKTYKCKN---------APYSG----YCV------
GL0417116 LASYKAGT-------------VVLQPK------DGKTYECKP---------FPYSG----YCK------
GL0772141 IASYKAGT-------------VVLQPK------DGKTYECKP---------FPYSG----YCV------
```

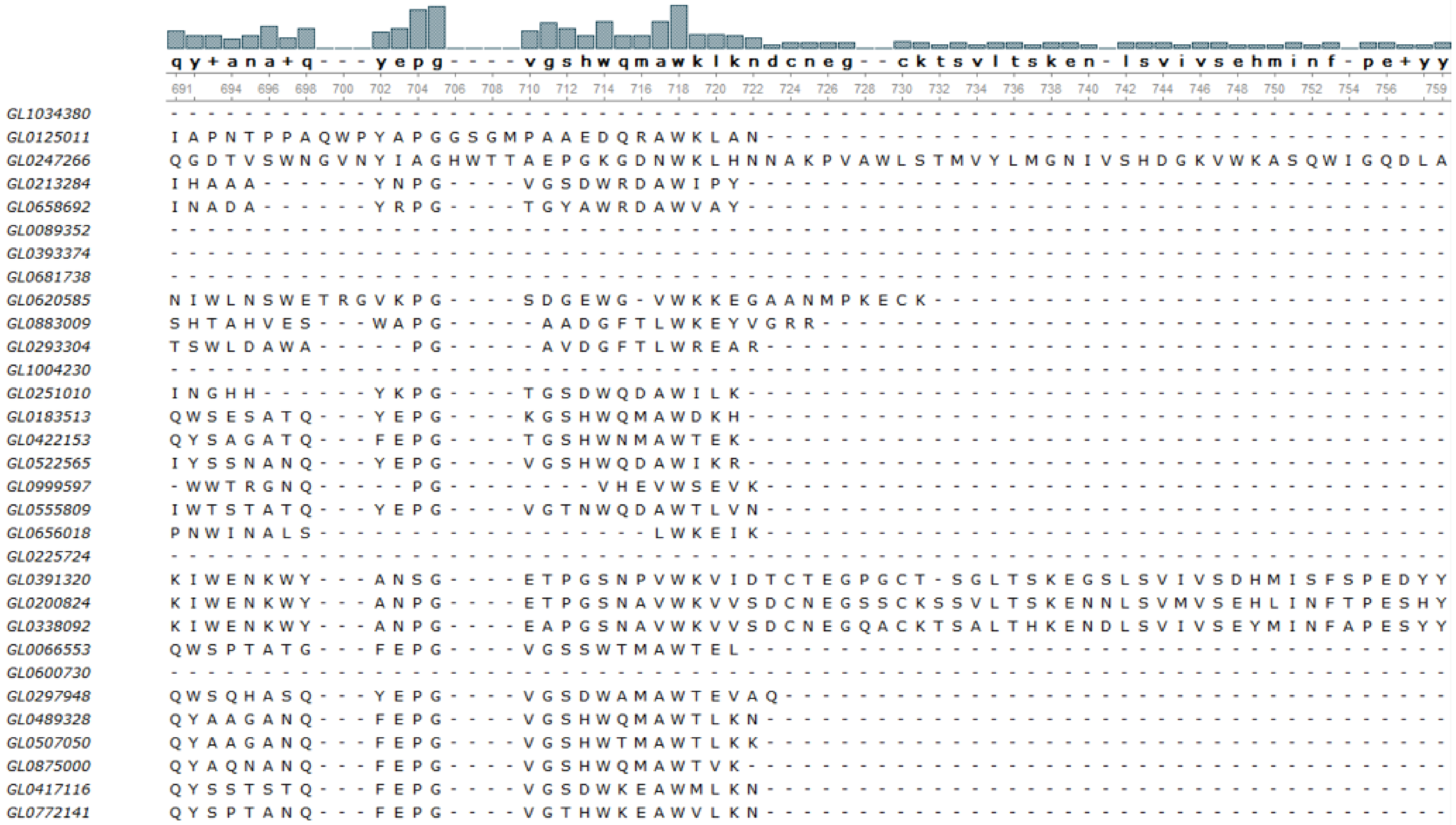

qy+ana+q---yepg----vgshwqmawklkndcneg--cktsvltsken-lsvivsehminf-pe+yy
691 694 696 698 700 702 704 706 708 710 712 714 716 718 720 722 724 726 728 730 732 734 736 738 740 742 744 746 748 750 752 754 756 759
GL1034380
GL0125011 IAPNTPPAQWPYAPGGSGMPAAEDQRAWKLAN
GL0247266 QGDTVSWNGVNYIAGHWTTAEPGKGDNWKLHNNAKPVAWLSTMVYLMGNIVSHDGKVWKASQWIGQDLA
GL0213284 IHAAA------YNPG----VGSDWRDAWIPY
GL0658692 INADA------YRPG----TGYAWRDAWVAY
GL0089352
GL0393374
GL0681738
GL0620585 NIWLNSWETRGVKPG----SDGEWG-VWKKEGAANMPKECK
GL0883009 SHTAHVES---WAPG-----AADGFTLWKEYVGRR
GL0293304 TSWLDAWA-----PG-----AVDGFTLWREAR
GL1004230
GL0251010 INGHH------YKPG----TGSDWQDAWILK
GL0183513 QWSESATQ---YEPG----KGSHWQMAWDKH
GL0422153 QYSAGATQ---FEPG----TGSHWNMAWTEK
GL0522565 IYSSNANQ---YEPG----VGSHWQDAWIKR
GL0999597 -WWTRGNQ-----PG--------VHEVWSEVK
GL0555809 IWTSTATQ---YEPG----VGTNWQDAWTLVN
GL0656018 PNWINALS------------------LWKEIK
GL0225724
GL0391320 KIWENKWY---ANSG----ETPGSNPVWKVIDTCTEGPGCT-SGLTSKEGSLSVIVSDHMISFSPEDYY
GL0200824 KIWENKWY---ANPG----ETPGSNAVWKVVSDCNEGSSCKSSVLTSKENNLSVMVSEHLINFTPESHY
GL0338092 KIWENKWY---ANPG----EAPGSNAVWKVVSDCNEGQACKTSALTHKENDLSVIVSEYMINFAPESYY
GL0066553 QWSPTATG---FEPG----VGSSWTMAWTEL
GL0600730
GL0297948 QWSQHASQ---YEPG----VGSDWAMAWTEVAQ
GL0489328 QYAAGANQ---FEPG----VGSHWQMAWTLKN
GL0507050 QYAAGANQ---FEPG----VGSHWTMAWTLKK
GL0875000 QYAQNANQ---FEPG----VGSHWQMAWTVK
GL0417116 QYSSTSTQ---FEPG----VGSDWKEAWMLKN
GL0772141 QYSPTANQ---FEPG----VGTHWKEAWVLKN

	gkisrvd-itphglqi-tf-np-knsmnisrlqsgiyfvki-ykdg-sitktirk
	760 764 766 768 770 772 774 776 778 780 782 784 786 788 790 792 794 796 798 800 802 804 806 808 810 814
GL1034380	---
GL0125011	---
GL0247266	PGQSELWKQQ---
GL0213284	---
GL0658692	---
GL0089352	---
GL0393374	---
GL0681738	---
GL0620585	---
GL0883009	---
GL0293304	---
GL1004230	---
GL0251010	---
GL0183513	---
GL0422153	---
GL0522565	---
GL0999597	---
GL0555809	---
GL0656018	---
GL0225724	---
GL0391320	GKMSRVDVINPAGIQMLSSVNPTKSSINISHLQAGIYFVRIHYKDGSSITKTIRK
GL0200824	GKISRVDIITPHGLQIMTFMNPGQNSMNISRLQSGIYFVKIQYKDGNSITKTIRK
GL0338092	NTISRVDLITPHGLQIITFTNPEKNSMNISRLQSGTYFVKILYKDGRSITKTIRK
GL0066553	---
GL0600730	---
GL0297948	---
GL0489328	---
GL0507050	---
GL0875000	---
GL0417116	---
GL0772141	---
