# Supplementary material for: Sequence and structure analyses of lytic polysaccharide monooxygenases mined from metagenomic DNA of humus samples around white-rot fungi in Cuc Phuong tropical forest, Vietnam
Source: PeerJ. 2024 Jun 24;12:e17553. doi: 10.7717/peerj.17553 (PMC11210479; doi:10.7717/peerj.17553)

Supplemental article S2: Prediction of spatial arrangement of the conservative amino acids in active sites of 31 putative LPMOs by the Alphafold2 and their comparison with the reference proteins of 8 phylogenetic groups by PyMOL. Magentas: Reference proteins in 8 phylogenetic groups (4OY6 for Group 1, 3UAM for Group 2, 5AA7 for Subgroup 3.1, 5LW4 for Subgroups 3.2 and 3.3, 2BEM for Subgroups 3.4, 3.5 and 3.6). Numbers indicate the positions of the active-site amino acids corresponding to H1-Hx-Fy motif in the reference proteins.

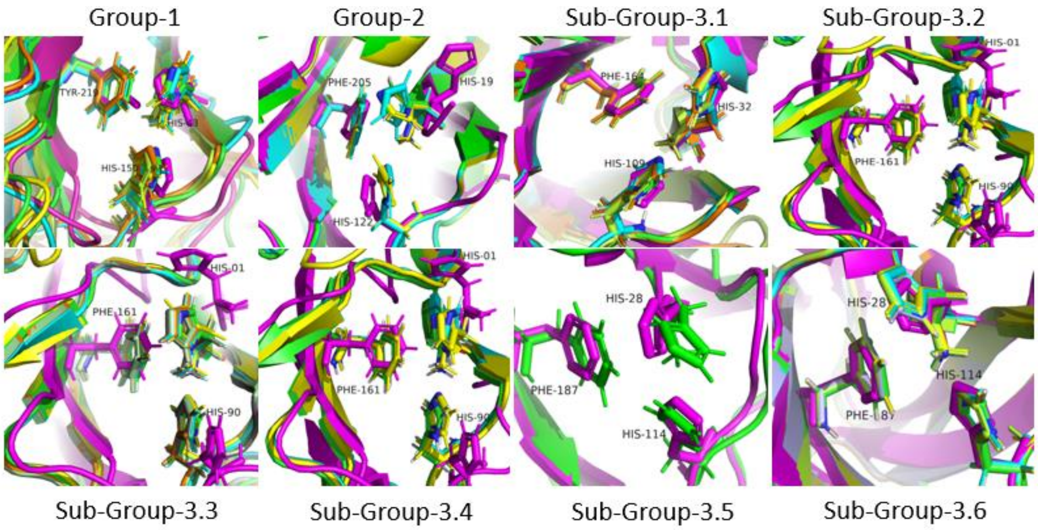

Supplement: Articlle S2 — Magentas: Reference proteins in 8 phylogenetic groups (4OY6 for Group 1, 3UAM for Group 2, 5AA7 for Subgroup 3.1, 5LW4 for Subgroups 3.2 and 3.3, 2BEM for Subgroups 3.4, 3.5 and 3.6). Numbers indicate the positions of the active-site amino acids corresponding to H1-Hx-Fy motif in the reference proteins [file peerj-12-17553-s006.pdf]
