# Supplementary material for: Sequence and structure analyses of lytic polysaccharide monooxygenases mined from metagenomic DNA of humus samples around white-rot fungi in Cuc Phuong tropical forest, Vietnam
Source: PeerJ. 2024 Jun 24;12:e17553. doi: 10.7717/peerj.17553 (PMC11210479; doi:10.7717/peerj.17553)
Supplement: Article S3 — A: Protein GL0247266; B: Protein GL0183513 [file peerj-12-17553-s007.pdf]

Supplemental article S3. Results of N-terminal amino acid sequencing by Edman degradation method. A: Protein GL0247266; B: Protein GL0183513.

A

Raw Pmol Table

	ASP	ASN	SER	GLN	THR	GLY	GLU	HIS	ALA	ARG	TYR	PRO	MET	VAL	TRP	PHE	ILE	LYS	LEU
1	5.559	2.004	4.342	1.677	2.065	14.33	2.290	10.10	9.613	4.887	1.157	2.496	0.579	1.963	0.629	3.033	1.090	1.131	1.651
2	5.536	2.594	3.882	2.357	3.593	38.83	2.825	7.245	11.92	4.295	2.167	6.660	1.251	4.751	1.202	2.917	1.517	1.760	3.555
3	7.090	3.120	5.306	3.234	6.167	28.53	4.617	5.565	38.47	4.558	2.991	7.502	2.949	8.583	1.614	3.315	2.885	3.333	5.805
4	9.320	4.319	6.288	5.052	9.323	26.60	7.573	4.705	36.53	5.791	4.854	8.954	16.07	13.46	2.557	4.259	4.765	7.008	8.600
5	11.45	5.990	7.866	5.635	12.57	27.26	17.71	3.809	36.34	6.143	6.360	10.44	15.48	18.58	2.877	5.703	6.766	10.66	11.48

Cycle	Residue	Amino Acid
**1**	blank	-
**2**	standard	
**3**	1	H
**4**	2	G
**5**	3	A
**6**	4	M
**7**	5	E

B

Raw Pmol Table

	ASP	ASN	SER	GLN	THR	GLY	GLU	HIS	ALA	ARG	TYR	PRO	MET	VAL	TRP	PHE	ILE	LYS	LEU
1	6.600	2.470	6.652	1.408	6.130	91.47	2.852	35.93	5.576	13.60	2.327	15.34	0.782	1.893	0.751	1.997	0.933	1.215	2.347
2	7.266	5.994	5.027	5.643	6.450	142.7	6.761	11.94	16.35	9.932	9.488	20.15	0.283	8.095	0.692	6.066	4.307	4.956	7.349
3	10.98	11.52	12.90	9.562	8.581	67.45	9.996	7.426	23.38	10.22	108.7	21.20	0.292	18.96	1.966	12.31	15.59	11.44	12.97
4	18.24	14.88	13.65	17.16	12.37	47.33	18.65	6.572	33.20	16.15	53.39	24.59	1.247	29.66	3.438	19.95	102.5	17.17	15.62
5	30.49	16.88	14.32	68.94	14.85	41.70	29.98	6.504	40.37	15.81	32.65	26.90	1.577	36.26	4.266	24.38	58.26	19.29	19.00

Cycle	Residue	Amino Acid
**1**	blank	-
**2**	standard	
**3**	1	H
**4**	2	G
**5**	3	Y
**6**	4	I
**7**	5	Q, (D, E)
